# The effectiveness of school-based obesity prevention interventions on the health behaviours of children aged 6–18 years: A secondary data analysis of a systematic review

**DOI:** 10.1016/j.pmedr.2025.103053

**Published:** 2025-03-31

**Authors:** Katrina McDiarmid, Tara Clinton-McHarg, Luke Wolfenden, Kate O'Brien, Daniel Chun Wei Lee, Ashleigh Stuart, Rebecca Kate Hodder

**Affiliations:** a National Centre of Implementation Science: Booth Building, Wallsend Health Services, Longworth Avenue, WALLSEND, NSW 2287, Australia; b School of Medicine and Public Health, College of Health Medicine and Wellbeing, University of Newcastle: University Dr, Callaghan, NSW 2308, Australia; c Melbourne School of Population and Global Health, Faculty of Medicine, Dentistry and Health Sciences, University of Melbourne: Parkville, VIC 3052, Australia; d Hunter Medical Research Institute: Lot 1 Kookaburra Cct, New Lambton Heights, NSW 2305, Australia

**Keywords:** Obesity, Child, School, Diet, Physical activity, Smoking, Alcohol

## Abstract

**Objectives:**

Obesity remains a contributor to the burden of disease globally. Suboptimal diet and physical inactivity are two rising risk factors of obesity in youth; both are targeted for obesity prevention. Further, these risk behaviours cluster and may be associated with other risks including smoking and alcohol intake. Few studies, however, have examined the extent to which interventions targeting obesity also impact on other health behaviours. The aim of this study is to synthesise the effects of child obesity prevention programs on diet, physical activity, tobacco smoking and alcohol intake, and to investigate differential effects by interventions that target different behaviours.

**Methods:**

A secondary data analysis of an existing systematic review was conducted. Literature searches identified any additional papers from 1990 to 2023 associated with the originally included studies. All papers were screened and were eligible if they reported any diet, physical activity, smoking or alcohol outcomes. Results for each health behaviour outcome were selected. Meta-analysis was conducted where possible to calculate standardised mean differences.

**Results:**

One hundred and four studies were eligible for inclusion. Fruit and vegetable intake (standardised mean difference (SMD) 0.104; 95 % (CI) (0.03, 0.17)), and sugar-sweetened beverage intake (SMD -0.126; 95 % CI (−0.22, −0.04)) were positively impacted by obesity prevention, as were physical activity (SMD 0.168; 95 % CI (0.05, 0.28)) and sedentary behaviour (SMD -0.021; 95 % CI (−0.03, −0.01)). Findings were mixed for tobacco smoking and alcohol intake.

**Conclusion:**

Independent of weight status, school obesity prevention programs may improve some measures of child dietary intake and physical activity.

**Study registration:**

Prospectively registered: PROSPERO: CRD42021281106.

## Highlights

• School obesity programs have a positive impact on diet and physical activity
• There is little evidence to assess the effects of programs on smoking and alcohol
• Little evidence exists of the effect of programs in secondary or high schools

## 1 Introduction

Obesity is a worldwide public health issue, with increasing prevalence across all age groups, increasing the burden of chronic diseases such as diabetes, cancers and cardiovascular diseases (World Health Organisation, 2021). In 2020, 175 million children and adolescents worldwide were living with obesity (Lobstein et al., 2023). The onset of obesity in childhood is of particular concern, as evidence suggests that children who are overweight or obese are more likely to live with overweight or obesity as adults (Lister et al., 2023). Therefore, preventing obesity in childhood is recommended by Australian and international guidelines to reduce the burden on individuals and the community (Langford et al., 2015).

Poor diet (excessive energy intake, low fruit and vegetable consumption) and physical inactivity (low activity and high sedentary behaviour levels) are the two primary modifiable risk factors for, and key behavioural targets to prevent, child obesity (World Health Organisation, 2014). Systematic reviews have also found these behaviours are associated with one another in adolescents (Laaksonen et al., 2001; Leech et al., 2014; Meader et al., 2016; Mello et al., 2021; Noble et al., 2015). Healthy dietary behaviours are positively associated with higher physical activity levels in adolescents, meaning that those who eat a healthy diet are more likely to be sufficiently active. Similarly, unhealthy dietary behaviours and physical inactivity (or sedentary behaviour) frequently co-occur (Laaksonen et al., 2001; Leech et al., 2014; Meader et al., 2016; Mello et al., 2021; Noble et al., 2015).

Other behavioural risks for chronic disease have been found to be associated with overweight and obesity. Tobacco smoking and excessive alcohol consumption are two such behaviours, (White et al., 2019; Kim et al., 2016) both leading to a range of lifestyle related cancers (Katzke et al., 2015). Initiation of these behaviours in adolescence leads to the sustainment in adulthood (Hale and Viner, 2016). Like diet and physical activity, smoking and alcohol consumption are also commonly associated with each other (Laaksonen et al., 2001; Meader et al., 2016; Noble et al., 2015), and evidence shows these four health risk behaviours cluster in adolescents. For example, a cross-sectional study of 853 Australian adolescents aged 18 years identified three main clusters of health risk behaviours: binge drinkers with poor diet (52 %); non-smokers with low physical activity and poor diet (24 %); and binge drinkers with poor diet who also smoke (24 %) (Champion et al., 2018). The presence of any two of these behaviours increases all-cause mortality in adulthood by 1.6 compared with the presence of one (Heroux et al., 2012).

Given the evidence demonstrating relationships between obesity and other health behaviours, it has been suggested that interventions targeting obesity and its key determinants, may also improve other health behaviours (Fleig et al., 2011). Recognising that many obesity-related health behaviours share similar neural circuitry (Baumeister and Vohs, 2007), behavioural change programs targeting one health behaviour may impact others. Demonstration of this relationship would provide more compelling evidence for obesity prevention initiatives, given their broader public health potential.

Schools are an attractive setting for obesity prevention interventions targeting children and adolescents, as they provide centralised access to students for prolonged periods. Schools also engage children and adolescents during a critical development period where health behaviours are being established. Further, supporting student health and wellbeing is an important part of a school's role addressed in their policies and infrastructure (Langford et al., 2015; Pulimeno et al., 2020). For example, many schools implement healthy canteen strategies to promote healthy eating and smoke-free grounds for everyone (Greenhalgh et al., 2022; Stylianou and Walker, 2018).

However, there are no current reviews of school-based obesity interventions examining their effects on multiple behaviours or synthesising the effects of different obesity intervention components. One recent systematic review of randomised controlled trials (RCTs) included 34 school-based interventions targeting two or more health behaviours, including, but not limited to, obesity prevention interventions (MacArthur et al., 2018). The authors synthesised impacts across a broad range of outcomes including healthy eating, physical activity, tobacco use, alcohol use, drug use, and mental health. However, there were not enough data available to determine the effects of the individual intervention components. The review concluded that some universal school interventions, especially those targeting multiple health behaviours, had positive effects on outcomes including substance use and physical activity. These findings suggest broader beneficial effects of obesity prevention interventions.

Given the absence of up-to-date systematic review evidence examining the effects of obesity-specific interventions on a range of lifestyle behaviours in children and adolescence, we aim to synthesise the effects of child obesity prevention programs that target a combination of healthy eating, physical activity, or both, on diet, physical activity, tobacco smoking and alcohol intake. We also aim to investigate differential effects by interventions that targeted diet, physical activity, or both. The results of this review will explore the impact of obesity prevention on lifestyle behaviours by demonstrating their benefits outside of weight management and will contribute to the evidence behind program development.

## 2 Material and methods

A secondary analysis of studies included in a recent systematic review assessing the effectiveness of childhood obesity prevention interventions on child weight status was undertaken (Hodder et al., 2022) to determine impact on diet, physical activity, tobacco smoking and alcohol intake. A protocol for this study was prospectively registered (PROSPERO: CRD42021281106) (McDiarmid et al., 2021). The study is exempt from ethical compliance.

### 2.1 Study inclusion criteria

The original review included RCTs comparing obesity prevention interventions targeting diet and/or physical activity behaviours, with a control group (no intervention or usual care) or another active intervention (i.e. head-to-head comparisons). For this secondary analysis, only RCTs comparing an obesity intervention to a control group published between 1990 and 2023 were included.

RCTs of interventions designed to treat (rather than prevent) childhood obesity or eating disorders or included any drug or surgery interventions; and those focussing on improving strength and fitness only (rather than to increase time in physical activity for preventing obesity) were excluded from the original review.

#### 2.1.1 Participants

Consistent with the original review, RCTs including children of any weight with a mean age between six and 18 years at baseline were eligible. Studies that only enrolled children who were overweight or obese at baseline (e.g. treatment rather than prevention), designed to prevent obesity in pregnant women, or children with a critical illness or severe co-morbidities, were excluded.

#### 2.1.2 Intervention

Consistent with the original review, eligible interventions were those that:

1. Were designed or had an underlying intention to prevent obesity (e.g. diet only, time in physical activity only, or both);
2. Had an active intervention period of any duration and reported follow-up outcome data at a minimum of 12 weeks from baseline;
3. Randomly assigned individuals (or groups) to an experimental group, however, for those with group randomisation, only cluster-RCTs (C-RCTs) with six or more groups were eligible; and
4. Were delivered by any personnel.

For the original review, interventions conducted in any setting were eligible for inclusion. For this analysis only studies conducted in the school setting were eligible, inclusive of kindergarten, primary, middle and secondary schools.

#### 2.1.3 Outcomes

To be included in the original review, studies were required to report both baseline and post-intervention data for one or more eligible obesity related outcomes, including body mass index (BMI)/zBMI score and prevalence of overweight and obesity.

To be eligible for this secondary data analysis, studies must have additionally reported an intervention effect on one or more of the following health behaviour outcomes that represent key contributors to the energy imbalance related to obesity: dietary intake (energy intake, fruit and vegetable intake, sugar-sweetened beverage intake), physical activity (physical activity (restricted to measures of intensity or duration), and time in sedentary behaviour), tobacco smoking (any), or alcohol consumption (any).

### 2.2 Search methods

The search strategy for the original review was implemented in electronic databases: (Cochrane Central Register of Controlled Trials, Medline, Embase, Cumulative Index to Nursing and Allied Health Literature and PsycINFO (Hodder et al., 2022). Studies were not excluded based on language.

All included studies and their associated records from the original review were screened in accordance with the updated eligibility criteria. Additional database searches (i.e. ClinicalTrials.gov, World Health Organisation International Clinical Trials Registry Platform) for associated trial registries were conducted to identify other potentially eligible associated records for each study. Authors of studies included in the previous review were contacted via email for any associated publications or relevant unpublished data prior to screening for eligible studies.

### 2.3 Study selection

Two review authors (KM, DCWL) independently screened full texts of studies from the original review, their associated records and any newly identified records using a standardised tool. Any differences were resolved through discussion, and if required, consultation with a third review author (RH).

### 2.4 Data collection

One review author (KM) independently extracted health behaviour outcome data (by group results and measures of effect) from included studies, using a standardised tool in REDcap (Harris et al., 2009). A second review author (KO) independently extracted the same data from a randomly selected 10 % of the included studies. Any differences in data extraction between review authors were resolved via discussion or with a third reviewer (RH). Authors of studies published with missing data were contacted to provide complete datasets. Any remaining studies with incomplete datasets were excluded from meta-analysis and synthesised narratively instead. Study characteristics (design, participants, interventions) were available from the original published review. We extracted information relevant to assessing risk of bias specific to the eligible outcomes of this study.

### 2.5 Assessment of risk of bias and quality of evidence

Risk of bias (ROB) of included studies was assessed using the first version of the Cochrane Handbook for Systematic reviews of Interventions risk of bias tool (Higgins and Altman, 2008). The ROB assessments from the original systematic review were available and adopted for this analysis for domains: sequence generation, allocation sequence concealment, blinding of participants and personnel, and other biases. The ROB for eligible health behaviour outcome specific domains were assessed for this analysis for the following domains: blinding of outcome assessment, incomplete outcome data, and selective outcome reporting. ROB was assessed independently by one review author (KM) with consolidation of 10 % with a second author (TCM) and summarised in tabular form. Any disagreements in ROB assessments were resolved via discussion, or consultation with a third reviewer (RH).

The Grading of Recommendations Assessment, Development and Evaluation (GRADE) approach was used to assess the quality of the evidence for each eligible health behaviour outcome. The GRADE assessment was completed for each outcome preferencing meta-analysis results, then results from synthesis without meta-analysis (SWiM), then for individual studies. The certainty of the body of evidence for each outcome was graded as ‘high’, ‘moderate’, ‘low’, or ‘very low’.

### 2.6 Data analysis and synthesis

Each outcome was synthesised by comparing the intervention to the control groups for all intervention types. Where multiple measures for an outcome were reported within a study, we used a decision hierarchy for selecting the single measure for inclusion in the review (Table 1, Table 2) (Wolfenden et al., 2022). The hierarchy was used to ensure the most rigorous outcome from each study was included. Where outcomes were divided into part days, preference was taken for school hours over other times. Where there were multiple measures of the same outcome (at the same level of the decision hierarchy), we randomly selected one via a random number generator. For each study reporting on multiple time points, data were extracted for the first follow up as per the methods of the original review. For tobacco smoking and alcohol outcomes, validated ‘current or daily use’ measures were preferred over ‘ever use’ measures.

**Table 1:** Decision hierarchy for diet and physical activity outcomes to be extracted for analysis.

	Level 1: Objective		Level 2: Subjective (validated tools as preference over non-validated)			
	Step 1	Step 2	Step 1	Step 2	Step 3	Step 4
Diet	Whole day estimates of diet including measures of the quantity or frequency of foods or energy intake	Part day (for example, during school hours) estimates of student diet	Whole day self-report estimates of diet including measures of the quantity or frequency of foods or energy intake	Part day (for example, during school hours) self-report estimates of student diet	Whole day proxy-report (for example, parent report) estimates of diet including measures of the quantity or frequency of foods or energy intake	Part day (for example, during school hours) proxy-report (for example, parent report) estimates of student diet
Physical activity	Device measured whole-day estimates of physical activity/sedentary behaviour including measures of intensity	Device measured part-day (for example, during school hours) estimates of physical activity/sedentary behaviour	Self-report whole-day estimates of physical activity/sedentary behaviour including measures of intensity	Self-report part-day (for example, during school hours) estimates of physical activity/sedentary behaviour	Proxy-report (for example, parent report) whole-day estimates of physical activity/sedentary behaviour including measures of intensity	Proxy-report (for example, parent report) part-day (for example, during school hours) estimates of physical activity/sedentary behaviour

**Table 2:** Decision hierarchy for tobacco smoking and alcohol outcomes to be extracted for analysis.

	Level 1	Level 2	Level 3
Tobacco smoking	Validated self-report measures of tobacco use	Non-validated self-report measures of tobacco use	Other estimates of tobacco use (e.g. cigarette butt counts)
Alcohol	Validated self-report measures of alcohol consumption	Non-validated self-report measures of alcohol consumption	Not applicable

Data were pooled in meta-analyses using a random effects model where at least two studies reporting the same outcome were identified. Standardised mean differences (SMD) and variances, as 95 % CI, were calculated to compare outcomes, as this allowed comparison of different outcome measures. For continuous outcomes, where reported, SMD's or model estimates of differences between groups at follow-up were extracted directly from the studies. Where model-based estimates were not available, group means, standard deviations and sample sizes were used. Where studies did not have group means or standard deviations, medians or inter-quartile ranges were used. Where studies reported change scores rather than follow-up results, follow-up results were imputed from the baseline values where possible, and baseline standard deviation was used as an estimate of follow-up standard deviation. Sensitivity analyses excluding studies with imputed values were conducted. For categorical outcomes, SMD's or odds ratios from models were used when provided. Where necessary, clustering was adjusted for by inflating the standard errors by multiplying by the square root of the design effect (Higgins et al., 2019). The design effect was calculated using (*DE* = 1 + (*m* – 1) × *ICC*), where m was the average cluster size and the intra-class coefficient (ICC) was either obtained from the study, or where it was not reported, the mean of those reported was used (ICC 0.06). Results from studies that had multiple intervention arms or where outcomes were reported by subgroup were combined.

Data not able to be pooled in meta-analyses were reported according to the Cochrane Handbook for Systematic Reviews of Intervention to synthesise results without meta-analysis (McKenzie and Brennan, 2019) and SWiM guidelines (Campbell et al., 2020). Where available, data were summarised using effect estimates as a first preference, followed by combined *p*-values. If neither of these were possible, vote counting was used based on direction of effect and results summarised using effect direction plots (Boon and Thomson, 2021). Individual study summaries were used when neither meta-analysis nor SWiM was unavailable, or when only one study was available for comparison.

When reporting, results from meta-analysis were prioritised as the most robust method of determining the intervention effect size and direction.

Effects were classified as small = 0.02, moderate = 0.5, or large = 0.8 (Cohen, 1987). I^2^, a measure of study heterogeneity, was reported and presence of heterogeneity (defined as I^2^ ≥ 50 %) explored in subgroup analysis by intervention type (described below). Exploration of further causes of heterogeneity, for example, age group, were outside the study scope.

Where possible, subgroup analysis was performed to identify any differential effects on each outcome by intervention type (e.g. diet only, physical activity only, or combined diet and physical activity). Meta-regression, was used to perform subgroup analysis as some subgroups had too few studies to measure variance precisely if modelled individually (Rubio-Aparicio et al., 2017). Results were presented as the pooled effect in each subgroup, with differences between subgroups tested using an omnibus test for subgroup differences. Meta-regression was only performed where the total number of studies pooled was greater than or equal to 10. Random effects meta-analysis was used to pool effect sizes and their variances for all outcomes using the R package ‘metafor’ (Viechtbauer, 2010).

## 3 Results

### 3.1 Study characteristics

**Fig. 1:**
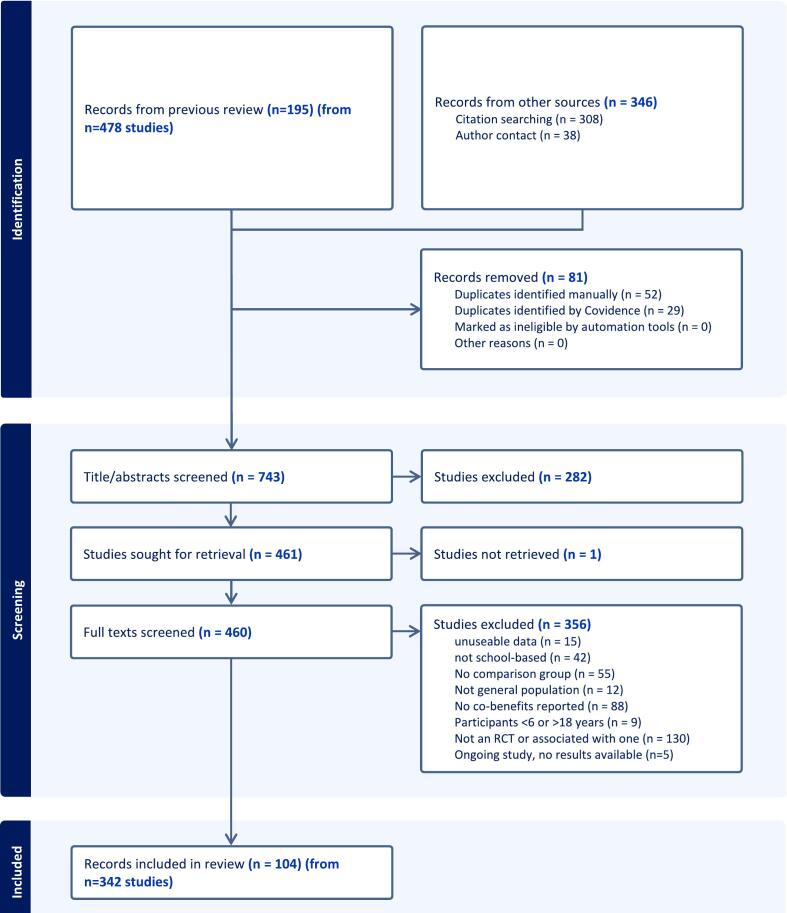
Flow of studies assessing other behaviour outcomes of school obesity prevention interventions from 1990 to 2023. RCT = randomised controlled trial.

Eight hundred and twenty-four unique records, including the 195 studies from *n* = 478 records from the original review, and the *n* = 346 potentially associated papers identified through the updated search and author contact, were screened against the updated eligibility criteria. One hundred and four records were eligible for inclusion from screening of 460 full texts (Fig. 1).

Of the 104 studies, 16 were RCTs and 88 were C-RCTs that compared one or more active intervention group to a control. A total of 10,2822 participants and 234 trial arms were analysed. All studies were conducted in schools whether during school time (*n* = 94) or as an after-school program (*n* = 10). Seventy-six were conducted in a primary school setting, 27 were conducted in a secondary school setting and one was conducted in both.

Participants mean age was 10.5 years, and most studies targeted both diet and physical activity. Intervention duration ranged from five weeks to four years.

Sixty-six studies reported a diet outcome, 88 reported a physical activity outcome, one reported a tobacco smoking outcome, and two reported an alcohol intake outcome [Appendix a: study characteristics].

### 3.2 Risk of Bias

Outcomes for most studies were deemed as ‘low’ ROB for random sequence generation (58 %; 144/249), ‘unclear’ for allocation concealment (61 %; 153/249), ‘high’ for blinding (67 %; 165/249), ‘low’ for incomplete outcome data (55 %; 137/247), ‘unclear’ for selective outcome reporting (86/246) and ‘low’ for other ROB (57 %; 142/249) [Appendix b: ROB results].

### 3.3 Diet outcomes

#### 3.3.1 Energy

**Overall effects:** Sixteen studies were synthesised that assessed energy intake. There was little to no difference in energy intake when all intervention types were combined (SMD -0.04; 95 % CI (−0.09,0.01); *n* = 16; participants = 11,380; moderate certainty evidence) [Table 3: Meta-analysis results]. One study assessed energy intake that was unable to be included in meta-analysis. This study reported a decrease in energy intake for both intervention arms in comparison to the control (Arm 1: mean difference: −0.39; Arm 2: mean difference: −0.18) (Williamson et al., 2012).

**Table 3:** Overall and subgroup meta-analysis results for diet and physical activity outcomes of randomised controlled obesity interventions published from 1990 to 2023.

Outcome	Subgroup	Studies	Participants	SMD (95 % confidence interval)	GRADE and direction of effect
Energy intake	All	16	11,380	-0.04 (−0.09, 0.01)	Moderate ▲
	Diet only	5	2248	−0.07 (−0.17, 0.02)	
	Diet and physical activity	12	15,896	−0.01 (−0.06, 0.03)	
Fruit and vegetable intake	All	39	36,983	0.1 (0.03, 0.17)*	Low ▲
	Diet only	7	3425	0.27 (0.11, 0.43)*	
	Physical activity only	2	736	0.04 (−0.32, 0.39)	
	Diet and physical activity	32	40,145	0.07 (0, 0.15)*	
Sugar-sweetened beverage intake	All	21	15,638	**−**0.13 (−0.22, −0.04)*	Low ▲
	Diet only	5	4942	0.02 (−0.14, 0.18)	
	Physical activity only	2	461	0.05 (−0.33, 0.42)	
	Diet and physical activity	16	13,929	**−**0.18 (−0.27, −0.08)*	
Physical activity	All	62	48,684	0.17 (0.05, 0.28)*	Low ▲
	Diet only	2	1123	0 (−0.64, 0.64)	
	Physical activity only	26	13,024	0.2 (0.03, 0.38)*	
	Diet and physical activity	41	38,635	0.18 (0.04, 0.32)*	
Sedentary behaviour	All	27	13,619	−0.02 (−0.03, −0.01)*	Moderate ▲
	Diet only	1	294	−0.14 (−1.39, 1.11)	
	Physical activity only	7	2585	0.08 (−0.03, 0.19)	
	Diet and physical activity only	21	12,619	−0.02 (−0.04, −0.01)*	

SMD = standardised mean difference, GRADE = Grading of Recommendations Assessment, Development and Evaluation, ▲ = positive direction of effect, * denotes a significant effect.

Energy intake, sugar-sweetened beverage intake and sedentary behaviour all displayed a reduction as a benefit. Fruit and vegetable intake, and physical activity all displayed as an increase as a benefit.

**Subgroup analysis:** Only interventions targeting diet alone, or a combination of diet and physical activity assessed energy intake. There was no differential effect in subgroup analysis by intervention target comparing diet alone and diet and physical activity interventions (*p*-value 0.26).

#### 3.3.2 Fruit and vegetable intake

**Overall effects** Thirty-nine studies were synthesised that assessed fruit and vegetable intake. A small increase in fruit and vegetable intake was found when intervention types were combined (SMD 0.1; 95 % CI (0.03, 0.17); *n* = 39, participants = 36,983; low certainty evidence). Ten studies assessed fruit and vegetable intake that were unable to be synthesised in meta-analysis and were assessed using the SWiM approach. There was evidence to suggest that obesity prevention interventions had a positive effect on fruit and vegetable intake with nine studies out of 10 (90 %) reporting an increase in intake in the intervention groups [Table 4: SWiM results].

**Table 4:** Synthesis Without Meta-analysis (direction of effect plot of intervention group) of select diet and physical activity outcomes from randomised controlled obesity interventions published from 1990 to 2023.

Study identification	Study design	No. participants: Intervention/control	Fruit and vegetable intake	Sugar-sweetened beverage intake	Physical activity	Sedentary behaviour
Andrade 2014	C-RCT	539/521				▲
Duncan 2019	C-RCT	311/278	▲	▲	▲	
Dunker 2018	C-RCT	131/139			▲	▲
Fairclough 2013	C-RCT	107/123	▲		▲	▲
Harrington 2018	C-RCT	735/626				▼
Ickovics 2019	C-RCT	482/113			◄►	
Magnusson 2012	C-RCT	103/82	▲			
Mauriello 2010	C-RCT	725/457	▲		▲	
Neumark-Sztainer 2003	C-RCT	84/106	▲	▼	▲	▼
Nyberg 2015	C-RCT	127/112	▲		▼	▲
Nyberg 2016	C-RCT	162/170	▼	▼	▼	▲
Pfeiffer 2019	C-RCT	753/766			▲	
Salmon 2008	C-RCT	213/55			▲	
Santos 2014	C-RCT	340/307			▲	
Siegrist 2018	C-RCT	243/191		▲		
Warren 2003	RCT	130/42	▲			
Wilksch 2015	C-RCT	347/473			▼	
Williamson 2012	C-RCT	1250/447			▼	▼
Xu 2015	C-RCT	605/503	▲	▼	▲	
Zota 2016	C-RCT	1609/2018	▲			

RCT = randomised controlled trial, C-RCT = cluster-randomised controlled trial.

▲ = positive direction of effect, ▼ = negative direction of effect, ◄► = no change/mixed effects/conflicting findings.

**Subgroup analysis:** There were no differential effects between groups (*p*-value 0.08).

#### 3.3.3 Sugar-sweetened beverage intake

**Overall effects** Twenty-one studies were synthesised that assessed sugar-sweetened beverage intake. A small decrease in sugar-sweetened beverage intake was found across studies of all intervention types (SMD -0.13; 95 % CI (−0.22, −0.04); *n* = 21; participants = 15,638; low certainty evidence). Five studies assessing sugar-sweetened beverage intake were unable to be synthesised in meta-analysis and were synthesised using the SWiM approach. There was evidence to suggest that obesity prevention interventions have a positive effect on sugar-sweetened beverage intake with three studies out of five (60 %) reporting a decrease in intake in the intervention group.

**Subgroup analysis:** There were no differential effects between groups (p-value 0.08).

### 3.4 Physical activity outcomes

#### 3.4.1 Physical activity

**Overall effects** Sixty-two studies assessed general physical activity including measures of physical activity intensity and total physical activity. Across all intervention types there was an increase in time spent in physical activity (SMD 0.17; 95 % CI (0.05, 0.28); *n* = 62; participants = 48,684; low certainty evidence). Fourteen studies assessing physical activity were not able to be included in meta-analysis and were synthesised using the SWiM approach. There is evidence to suggest obesity prevention programs positively impact physical activity levels as nine out of 14 (64 %) studies reported an increase in physical activity in the intervention groups. Two studies were unable to be synthesised in meta-analysis or by the SWiM approach. One study reported an increase in moderate-vigorous physical activity in the intervention group compared to control (Martinez-Vizcaino, 2014). The other study reported no difference between groups nor a direction of effect for the intervention group (Telford, 2012).

**Subgroup analysis:** There were no differential effects between the interventions (*p*-value 0.83).

#### 3.4.2 Sedentary behaviour

**Overall effects** Twenty-seven studies assessed sedentary behaviour. There was a small reduction in sedentary behaviour (SMD -0.02; 95 % CI (−0.03, −0.01); *n* = 27; participants = 13,619; moderate certainty evidence). Eight studies assessing sedentary behaviour were not able to be included in meta-analysis and were synthesised using the SWiM approach. There is evidence to suggest that obesity prevention interventions have a negative effect on sedentary behaviour as five out of eight studies (63 %) reported an increase in sedentary behaviour in the intervention groups. One study was unable to be synthesised in meta-analysis or by the SWiM approach but reported an increase in sedentary time in the intervention group as compared with the control (Martinez-Vizcaino, 2014).

**Subgroup analysis:** There were no differential effects between groups by intervention type (*p*-value 0.18).

### 3.5 Tobacco

The one study that assessed tobacco smoking found no difference in the intervention group compared to control group with an adjusted odds ratio of 1.0 (95 % CI (0.3, 4.1) (low certainty evidence).

### 3.6 Alcohol

The two studies that assessed alcohol consumption both targeted combined diet and physical activity and reported mixed effects. One trial did not report baseline figures thus the direction of effect could not be determined, however the intervention group reported significantly less alcohol intake than the control group at post intervention (Chi-square 4.28; p-value 0.04) (Melnyk, 2013). The other study reported an increase in alcohol intake across all intervention and control groups but it was unclear whether there was a difference between groups (Lana, 2014) (very low certainty evidence).

## 4 Discussion

This study examined the effects of school-based obesity prevention interventions on diet, physical activity, tobacco smoking and alcohol intake outcomes. Broadly, the review found school-based obesity prevention interventions may have a small positive effect on fruit and vegetable intake, sugar-sweetened beverage intake, physical activity, and sedentary behaviour, but not energy intake. There were too few studies to assess the effects of school-based obesity prevention interventions on tobacco or alcohol use behaviours.

While we found no significant subgroup interactions, obesity prevention interventions improved diet outcomes when they included a dietary component. For example, fruit and vegetable intake were positively impacted by diet only interventions, or combined diet and physical activity interventions. For both, the effect sizes for the physical activity only intervention subgroup were markedly smaller. The finding that obesity prevention interventions that included dietary components appear more likely to impact dietary outcomes is consistent with other reviews (Singhal et al., 2021; Nury et al., 2022). Nutritional intake can be influenced by many different interventional strategies which were reflected in the studies included in this review. Examples include directly changing the food environment by increasing provision of fruits and vegetables and increasing nutritional education within the curriculum, both of which are proven strategies to improve the quality of nutritional intake (Nury et al., 2022; Micha et al., 2018). The findings suggests that obesity prevention interventions that are seeking to achieve co-benefits on diet outcomes should include components specifically targeting this outcome.

Intake of sugar-sweetened beverages was improved in the combined diet and physical activity subgroup but not diet only. This is consistent with a recent systematic review that reported little to no difference in sugar-sweetened beverage intake following school-based nutrition interventions to prevent obesity (Nury et al., 2022). This could be attributed to the types of dietary behaviours targeted within these interventions since their primary target was to prevent obesity. This contrasts with another systematic review that found school-based interventions to specifically reduce sugar-sweetened beverage intake to be successful among adolescents (Vézina-Im et al., 2017). It could be that the lack of effect found in the studies analysed here that targeted diet alone may not have focused specifically on sugar-sweetened beverage reduction enough to warrant an effect.

Obesity prevention interventions also improved physical activity outcomes overall. Obesity prevention interventions targeting physical activity aim to increase physical activity levels, often by increasing the environmental exposure and increasing knowledge of the benefits of physical activity (Neil-Sztramko et al., 2021). Within schools, this often refers to a change in curriculum, or increased opportunities to participate in physical activity within class time or break time (Neil-Sztramko et al., 2021). We found physical activity was positively impacted by obesity prevention interventions that targeted physical activity only, or combined diet and physical activity; but not interventions targeting dietary behaviours only. The finding is also consistent with a previous review in primary schools (Singhal et al., 2021) however it contrasts with another similar review (Mead et al., 2017). On measures of sedentary behaviour, however, such patterns of subgroup effects were not apparent. Overall and for all subgroups, effects sizes were very small, though significant for the diet and physical activity combined subgroup. Potentially, effects may have been larger among subgroups that included strategies directly targeting sedentary behaviour (and not physical activity broadly). Further research is required to confirm this hypothesis. It is also possible that a physical activity intervention alone, which has been shown to increase physical activity may also show an increase in sedentary behaviour as a compensatory action (Ridgers et al., 2014).

Perhaps the most notable finding of the review is the lack of primary trials that have explored the effects of obesity prevention interventions on other health behaviours. We found just one of the 104 included studies assessed tobacco smoking and two assessed alcohol. As a result, the potential beneficial effects of obesity related interventions on these behaviours are not known. Previous studies have reported that improving diet and physical activity behaviours improves substance use behaviours providing a strong basis to hypothesise that the effects of obesity prevention interventions may also extend to substance use (Meader et al., 2016; Padrão et al., 2007). For example, increased physical activity reduced weekly alcohol consumed in adults (Hajat et al., 2019) and had a higher likelihood of attempting to quit smoking (Linke et al., 2016). Nonetheless, the findings of our review identify a significant evidence gap and an opportunity for future research. Such research may provide evidence of the potential for obesity prevention initiatives in schools to address other pressing public health challenges.

A limitation of this review is that the certainty of the evidence ranged from very low to moderate with most of the reported outcomes classified as low-certainty evidence as per the GRADE approach. As such there may be a small positive effect on fruit and vegetable intake, sugar-sweetened beverage intake, physical activity and sedentary behaviour. It is uncertain whether tobacco smoking and alcohol is influenced positively or negatively through school-based obesity interventions. Future high-quality studies are needed to increase the certainty of the evidence. While in many instances effects were positive, effect sizes were small for each of the outcomes investigated.

Most included studies were from a primary school setting and as such involved participants aged six to 12 years; and limits the generalisability of these results to this age group. Additionally, all studies were from middle-high income countries which limits the generalisability of our findings to other populations, regions and socioeconomic contexts. Future reviews could investigate more thoroughly the equity aspects of included studies (Nobles et al., 2021). Overall, this review was limited to the sample and measurement tools used in each of the included studies. Considering that this review included only those studies with a focus on obesity, there is also capacity for new research to include studies that target these behaviours without obesity being the primary outcome of interest, which may extend the range of included studies to those that assess other health behaviours such as sleep, tobacco and alcohol.

## 5 Conclusions

School-based child obesity prevention programs may have a small positive effect on other chronic disease risks, including diet and physical activity outcomes. Future research, in particular the generation of primary studies should prioritise examination of their effects on substance use behaviours. Such evidence may provide greater insight into the relationship between these risks and enable better quantification of the potential health benefits of obesity prevention initiatives.

## CRediT authorship contribution statement

**Katrina McDiarmid:** Writing – review & editing, Writing – original draft, Visualization, Project administration, Methodology, Investigation, Funding acquisition, Formal analysis, Data curation, Conceptualization. **Tara Clinton-McHarg:** Writing – review & editing, Supervision, Methodology, Formal analysis, Conceptualization. **Luke Wolfenden:** Writing – review & editing, Writing – original draft, Supervision, Methodology, Conceptualization. **Kate O'Brien:** Writing – review & editing, Supervision, Resources, Methodology, Formal analysis, Data curation. **Daniel Chun Wei Lee:** Writing – review & editing, Methodology. **Ashleigh Stuart:** Writing – review & editing, Software, Methodology, Formal analysis, Data curation. **Rebecca Kate Hodder:** Writing – review & editing, Writing – original draft, Visualization, Supervision, Resources, Methodology, Formal analysis, Data curation, Conceptualization.

## Funding

Funding support for statistical analysis provided by the Hunter Medical Research Institute Clinical Research Design & Statistics team. KM is supported through The University of Newcastle Research Training Scheme scholarship and stipend. The study funder had no role in the study design, data collection, data analysis, data interpretation, or writing of the report.

## Declaration of competing interest

The authors declare that they have no known competing financial interests or personal relationships that could have appeared to influence the work reported in this paper.

## Data Availability

Data will be made available on request.

## Appendix A Supplementary data

Supplementary material 1 Image 1

Supplementary material 2 Image 2
