# Supplementary material for: The effectiveness of school-based obesity prevention interventions on the health behaviours of children aged 6–18 years: A secondary data analysis of a systematic review
Source: Prev Med Rep. 2025 Mar 31;53:103053. doi: 10.1016/j.pmedr.2025.103053 (PMC11999465; doi:10.1016/j.pmedr.2025.103053)
Supplement: Supplementary file 1 — Supplementary material 1 [file mmc1.docx]

**Appendix a**

**Table S1.** Characteristics of included studies assessing school obesity prevention interventions in children from 1990-2023

| Author year  Country | Study characteristics  Design (cluster type)  Setting  Age group years (mean age)  Gender | Number of participants (overall) | | Intervention  Treatment arms  Targeted behaviour  Theory  Duration | Comparator | Outcomes  Health behaviour outcomes (italicised text denotes extracted outcome) |
| --- | --- | --- | --- | --- | --- | --- |
|  |  | Randomised | Analysed |  |  |  |
| Adab 2018  United Kingdom | Design: C-RCT (School)  Setting: School * + Home + Community  Age group: 6-12 (6·3)  Gender: mixed | 2462 | 837 | Arms: 1  Target: DPA  Theory: not reported  Duration: ≤ 12 months | Control: usual practice | Diet:   - *Energy* - *Fruit and vegetable intake*   Physical activity:   - *MVPA* - *Sedentary behaviour* |
| Amaro 2006  Italy | Design: C-RCT (Classroom)  Setting: School  Age group: 6-12 (12·4)  Gender: mixed | 291 | 241 | Arms: 1  Target: Diet  Theory: not reported  Duration: ≤ 12 months | Control: no intervention | Diet:   - *Vegetable intake*   Physical activity:   - *Daily physical activity* |
| Andrade 2014  Ecuador | Design: C-RCT (School)  Setting: School  Age group: 13-18 (intv = 12·8, control = 12·9)  Gender: mixed | 1440 | 1060 | Arms: 1  Target: DPA  Theory: SCT, IMB model, Control theory, TTM and TPB  Duration: > 12 months | Control: usual care | Diet:   - *Fruit and vegetable intake*   Physical activity:   - *Sedentary behaviour* |
| Arlinghaus  2021  USA | Design: RCT  Setting: School  Age group: 6-12 (weekday 12·10, weekend 12·06)  Gender: mixed | 491 | 329 | Arms: 1  Target: physical activity  Theory: SCT  Duration: ≤ 12 months | Control: usual care | Physical activity:   - *Sedentary behaviour* |
| Barbeau 2007  USA | Design: RCT  Setting: School (ASP)  Age group: 6-12 (9·5)  Gender: Girls only | 309 | 201 | Arms: 1  Target: physical activity  Theory: not reported  Duration: ≤ 12 months | Control: no intervention | Physical activity:   - *MVPA* |
| Bohnert 2013  USA | Design: RCT  Setting: School (ASP)  Age group: 6-12 (intv = 9·02, control = 9·38)  Gender: Girls only | 133 | 76 | Arms: 1  Target: DPA  Theory: SCT and Sociocultural theory  Duration: ≤ 12 months | Control: No intervention | Diet:   - *Vegetable intake*   Physical activity:   - *Total physical activity* |
| Bonsergent 2013  France | Design: C-RCT (School)  Setting: School* + Health Service + Community  Age group: 13-18 (15·8)  Gender: mixed | 5354 | 3538 | Arms: 3  Target: DPA  Theory: not reported  Duration: > 12 months | Control: No intervention | Physical activity:   - *Global physical activity* - Vigorous physical activity - Moderate physical activity |
| Brito Beck da Silva 2019  Brazil | Design: C-RCT (School)  Setting: School* + Home  Age group: 13-18 (14·5)  Gender: mixed | 895 | 602 | Arms: 1  Target: DPA  Theory: CBT  Duration: ≤ 12 months | Control: Waitlist | Diet:   - *Fruit intake* - *Soft drink intake* |
| Caballero 2003  USA | Design: C-RCT (School)  Setting: School* + Home  Age group: 6-12 (7·6)  Gender: mixed | 1714 | 1409 | Arms: 1  Target: DPA  Theory: SLT, and principles of American Indian culture and  practice  Duration: > 12 months | Control: Usual care presumed (no details provided but school-based  Intervention) | Diet:   - *Energy intake*   Physical activity:   - *Total physical activity* |
| Christiansen 2013  Denmark | Design: C-RCT (School)  Setting: School + Community  Age group: 13-18 (12·6)  Gender: mixed | 1348 | 989 | Arms: 1  Target: physical activity  Theory: Social Ecological  Duration: > 12 months | Control: Usual care | Physical activity:   - Overall physical activity - *MVPA* - *Sedentary time* |
| Clemes 2020  United Kingdom | Design: C-RCT (School)  Setting: School  Age group: 6-12 years (9·3)  Gender: mixed | 176 | 168 | Arms: 1  Target: physical activity  Theory: COM-B with BCW, TDF  Duration: ≤ 12 months | Control: Usual practice | Physical activity:   - Light physical activity - *MVPA* - *Sedentary behaviour* |
| Cunha 2013  Brazil | Design: C-RCT (Classroom)  Setting: School* + Home  Age group: 6-12 (intv = 11·2, control = 11·2)  Gender: mixed | 574 | 559 | Arms: 1  Target: Diet  Theory: TTM  Duration: ≤ 12 months | Control: No intervention | Diet:   - *Fruit intake* - Vegetable intake - Soft drink intake - *Juice intake* |
| Damsgaard 2014  Denmark | Design: C-RCT - crossover (School)  Setting: School  Age group: 6-12 (10·0)  Gender: mixed | 823 | 823 | Arms: 1  Target: Diet  Theory: not reported  Duration: ≤ 12 months | Control: Usual care (packed lunch from home) | Diet:   - *Energy* - Fruit intake - *Vegetable intake* |
| Davis 2021  USA | Design: C-RCT (School)  Setting: School  Age group: 6-12 (9·23)  Gender: mixed | 3135 | 3135 | Arms: 1  Target: Diet  Theory: Social ecological-transactional model  Duration: ≤ 12 months | Control: Delayed intevention | Diet:   - *Energy* - Fruit intake - *Vegetable intake* - *Sugar-sweetened beverage intake* |
| Dewar 2013  Australia | Design: C-RCT (School)  Setting: School* + Home  Age group: 13-18 (intv = 13·20, control = 13·15)  Gender: Girls only | 357 | 294 | Arms: 1  Target: DPA  Theory: SCT  Duration: ≤ 12 months | Control: Usual care presumed as no details but schoolbased intervention | Diet:   - *Energy*   Physical activity:   - *Accelerometer counts* - MVPA - Vigorous physical activity |
| Donnelly 2009  USA | Design: C-RCT (School)  Setting: School  Age group: 6-12 (Grade 2: intv female = 7·7, control female = 7·8; intv male = 7·7, control male = 7·8. Grade 3: intv female = 8·7, control female = 8·7; intv male = 8·7, control male = 8·8)  Gender: mixed | 1527 | 1490 | Arms: 1  Target: physical activity  Theory: not reported  Duration: > 12 months | Control: Usual care - regular classroom instruction without  physically active lessons | Physical activity:   - *Accelerometer counts* - MVPA |
| Drummy 2016  Northern Ireland | Design: C-RCT (Classroom)  Setting: School  Age group: 6-12 (9·5)  Gender: mixed | 120 | 107 | Arms: 1  Target: physical activity  Theory: not reported  Duration: ≤ 12 months | Control: Usual practice | Physical activity:   - *MVPA* |
| Duncan 2019  New Zealand | Design: C-RCT (School)  Setting: School* + Home  Age group: 6-12 (intv = 8·71, control = 8·74)  Gender: mixed | 675 | 589 | Arms: 1  Target: DPA  Theory: SCT, theory of reasoned action and planned behaviour  Duration: ≤ 12 months | Control: Waitlist | Diet:   - *Fruit intake* - Vegetable intake - *Soft drink intake*   Physical activity:   - *Accelerometer counts* |
| Dunker 2018  Brazil | Design: C-RCT (School)  Setting: School (ASP)* + Home  Age group: 13-18 (13·39)  Gender: Girls only | 270 | 270 | Arms: 1  Target: DPA  Theory: SCT  Duration: ≤ 12 months | Control: Usual practice | Diet:   - *Fruit intake* - Vegetable intake - Soft drink intake - *Artificial juice intake*   Physical activity:   - *Daily physical activity* - *Sedentary behaviour* |
| Ezendam 2012  Netherlands | Design: C-RCT (School)  Setting: School  Age group: 13-18 (intv = 12·7, control = 12·6)  Gender: mixed | 883 | 676 | Arms: 1  Target: DPA  Theory: TPB, Precaution Adoption Process Model, Implementation intentions  Duration: ≤ 12 months | Control: No intervention | Diet:   - *Fruit intake* - Vegetable intake - *Sugar-sweetened beverage intake*   Physical activity:   - *Accelerometer count* |
| Fairclough 2013  United Kingdom | Design: C-RCT (School)  Setting: School  Age group: 6-12 (intv = 10·6, control = 10·7)  Gender: mixed | 318 | 230 | Arms: 1  Target: DPA  Theory: SCT  Duration: ≤ 12 months | Control: Did not teach a specific unit focused on healthy  eating and physical activity | Diet:   - Fruit intake - *Vegetable intake*   Physical activity:   - *Light physical activity* - Moderate physical activity - Vigorous physical activity - Sedentary behaviour |
| Farmer 2017  New Zealand | Design: C-RCT (School)  Setting: School  Age group: 6-12 (intv = 8.0, control = 7·9)  Gender: mixed | 902 | 715 | Arms: 1  Target: physical activity  Theory: not reported  Duration: ≤ 12 months | Control: Usual practice | Physical activity:   - *Accelerometer counts* - MVPA |
| Foster 2008  USA | Design: C-RCT (School)  Setting: School  Age group: 6-12 (intv = 11·13, control = 11·2)  Gender: mixed | 1349 | 843 | Arms: 1  Target: DPA  Theory: settings-based approach; CDC Guidelines to Promote Lifelong  Healthy Eating and physical activity  Duration: > 12 months | Control: No intervention | Diet:   - *Energy* - *Fruit and vegetable intake*   Physical activity:   - *Total physical activity* - *Sedentary behaviour* |
| Gentile 2009  USA | Design: C-RCT (School)  Setting: School* + Community + Home  Age group: 6-12 (intv = 9·6, control = 9·6)  Gender: mixed | 1323 | 1201 | Arms: 1  Target: DPA  Theory: SEM  Duration: ≤ 12 months | Control: Community component only | Diet:   - *Fruit and vegetable intake*   Physical activity:   - *Accelerometer counts* |
| Gortmaker 1999a  USA | Design: C-RCT (School)  Setting: School + Home  Age group: 6-12 (11·7)  Gender: Girls only | 1560 | 1295 | Arms: 1  Target: DPA  Theory: Behavioural Choice and SCT  Duration: > 12 months | Control: Usual care, health curricula and PE classes | Diet:   - *Energy* - *Fruit and vegetable intake*   Physical activity:   - *MVPA* |
| Grydeland 2014  Norway | Design: C-RCT (School)  Setting: School* + Home  Age group: 6-12 (intv = 11·2, control = 11·2)  Gender: mixed | 2165 | 1361 | Arms: 1  Target: DPA  Theory: SEM  Duration: > 12 months | Control: Usual care presumed as no details but school-based  intervention | Diet:   - Fruit intake - *Vegetable intake* - *Soft drink intake* - Fruit drink intake   Physical activity:   - Total physical activity - Light physical activity - *MVPA* - *Sedentary behaviour* |
| Habib-Mourad 2014  Lebanon | Design: C-RCT (School)  Setting: School* + Home  Age group: 6-12 (intv = 10·39, control = 10·1)  Gender: mixed | 374 | 363 | Arms: 1  Target: DPA  Theory: SCT  Duration: ≤ 12 months | Control: Usual curriculum | Diet:   - *Fruit intake* - *Soft drink intake* - Sugar-sweetened beverage intake   Physical activity:   - *Recess activity* |
| Habib-Mourad 2020  Lebanon | Design: C-RCT (School)  Setting: School* + Home  Age group: 6-12 (9·95)  Gender: mixed | 1239 | 974 | Arms: 1  Target: DPA  Theory: SCT  Duration: > 12 months | Control: Waitlist | Diet:   - *Fruit intake* - Vegetable intake - *Soft drink intake* - Sugar-sweetened beverage intake   Physical activity:   - *After-school physical activity* |
| Haerens 2006  Belgium | Design: C-RCT (School)  Setting: School* + Home  Age group: 13-18 (13·1)  Gender: mixed | 2840 | 2291 | Arms: 1  Target: DPA  Theory: an ecological framework  Duration: > 12 months | Control: Usual care presumed as no details but school-based  intervention | Diet:   - *Fruit intake* - *Water intake* - Soft drink intake   Physical activity:   - Light physical activity - *MVPA* - *Sedentary behaviour* |
| Harrington 2018  United Kingdom | Design: C-RCT (School)  Setting: School  Age group: 13-18 (12·8)  Gender: Girls only | 1753 | 1361 | Arms: 1  Target: physical activity  Theory: SCT  Duration: ≤ 12 months | Control: Usual practice | Physical activity:   - *MVPA* - Average acceleration - Light physical activity - *Sedentary behaviour* |
| HEALTHY Study Group 2010  USA | Design: C-RCT (School)  Setting: School  Age group: 6-12 (intv = 11·3, control = 11·3)  Gender: mixed | 6413 | 4603 | Arms: 1  Target: DPA  Theory: not reported  Duration: > 12 months | Control: No intervention - assessment only | Diet:   - *Energy* - *Fruit and vegetable intake* |
| Herscovici 2013  Argentina | Design: C-RCT (School)  Setting: School  Age group: 6-12 (intv = 9·64, control = 9·76)  Gender: mixed | 405 | 369 | Arms: 1  Target: DPA  Theory: not reported  Duration: ≤ 12 months | Control: Usual care presumed as no details but school-based  intervention | Diet:   - Fruit intake - *Vegetable intake* - Soft drink intake - *Sugar-sweetened beverage intake* - Juice intake |
| Hollis 2016  Australia | Design: C-RCT (School)  Setting: School* + Community + Home  Age group: 6-12 (12·0)  Gender: mixed | 1233 | 985 | Arms: 1  Target: physical activity  Theory: SCT and socio-ecological theory  Duration: > 12 months | Control: Usual practice | Physical activity:   - MVPA - Vigorous physical activity - *Moderate physical activity* |
| Howe 2011  USA | Design: RCT  Setting: School (ASP)  Age group: 6-12 (9·75)  Gender: Boys only | 106 | 106 | Arms: 1  Target: physical activity  Theory: not reported  Duration: ≤ 12 months | Control: No intervention and were not allowed to stay for  the after-school intervention but rather instructed  not to change their daily after-school routine | Physical activity:   - *MVPA* |
| Ickovics 2019  USA | Design: C-RCT (School)  Setting: School* + Community + Home  Age group: 6-12 (10·9)  Gender: mixed | 756 | 595 | Arms: 3  Target: Diet, physical activity, DPA  Theory: not reported  Duration: > 12 months | Control: Attention control - delayed intervention schools health-focused messages not related with obesity prevention were implemented | Physical activity:   - *Total physical activity* |
| James 2004  United Kingdom | Design: C-RCT (Classroom)  Setting: School  Age group: 6-12 (8·7)  Gender: mixed | 644 | 574 | Arms: 1  Target: Diet  Theory: not reported  Duration: ≤ 12 months | Control: Usual care presumed as no details but school-based  intervention | Diet:   - *Carbonated drink intake* - Water intake |
| Kennedy 2018  Australia | Design: C-RCT (School)  Setting: School* + Home  Age group: 13-18 (14·1)  Gender: mixed | 607 | 600 | Arms: 1  Target: physical activity  Theory: SCT and social-determination theory  Duration: ≤ 12 months | Control: Waitlist | Physical activity:   - *MVPA* |
| Kipping 2014  United Kingdom | Design: C-RCT (School)  Setting: School* + Home  Age group: 6-12 (9·5)  Gender: mixed | 2221 | 1825 | Arms: 1  Target: DPA  Theory: SCT  Duration: ≤ 12 months | Control: Standard teaching | Diet:   - *Fruit and vegetable intake*   Physical activity:   - *MVPA* - *Sedentary behaviour* |
| Kobel 2017  Germany | Design: C-RCT (Classroom)  Setting: School* + Home  Age group: 6-12 (7·1)  Gender: mixed | 525 | 479 | Arms: 1  Target: DPA  Theory: Bandura’s social cognitive theory  Duration: ≤ 12 months | Control: Regular school curriculum | Diet:   - *Fruit and vegetable intake* - *Soft drink intake*   Physical activity:   - *MVPA* |
| Kocken 2016  The Netherlands | Design: C-RCT (School)  Setting: School* + Home  Age group: 6-12 (intv = 9·2, control = 9·1)  Gender: mixed | 1112 | 790 | Arms: 1  Target: DPA  Theory: TPB, Behavior change theory  Duration: ≤ 12 months | Control: Usual curriculum | Diet:   - *Energy* - Fruit intake - *Vegetable intake* - *Sugar-sweetened beverage intake* - Fruit drink intake   Physical activity:   - Accelerometer counts - *MVPA* - *Sedentary behaviour* |
| Kriemler 2010  Switzerland | Design: C-RCT (School)  Setting: School* + Home  Age group: 6-12 (1st graders = 6·9; 5^th^ graders intv = 11·0, control = 11·3)  Gender: mixed | 502 | 502 | Arms: 1  Target: physical activity  Theory: SEM  Duration: ≤ 12 months | Control: Not informed of an intervention group | Physical activity:   - *Total physical activity* - MVPA |
| Kubik 2021  USA | Design: RCT  Setting: School (ASP)* + Home  Age group: 6-12 (9·3)  Gender: mixed | 132 | 122 | Arms: 1  Target: DPA  Theory: Social–ecological framework, healthy learner model for student chronic condition management  Duration: ≤ 12 months | Control: Newsletter only | Diet:   - *Energy* - *Fruit intake* - Vegetable intake - *Sugar-sweetened vegetable intake* - Juice intake   Physical activity:   - Moderate physical activity - *Light physical activity* - Vigorous physical activity - *Sedentary behaviour* |
| Lana 2014  Spain and Mexico | Design: RCT  Setting: School* + Home  Age group: 13-18 (intv: 26·6% = 12, 38·5% = 13, 25·7% = 14, 9·2% = ≥15; control: 20·5% = 12, 42·7% = 13, 27·4% = 14, 9·4% = ≥15)  Gender: mixed | 2001 | 737 | Arms: 1  Target: DPA  Theory: ASE, TTM  Duration: ≤ 12 months | Control: No intervention presumed as no details | Diet:   - *Fruit intake* - Vegetable intake   Physical activity:   - *Sedentary behaviour* |
| Leme 2016  Brazil | Design: C-RCT (School)  Setting: School* + Home  Age group: 13-18 (16·05)  Gender: Girls only | 253 | 194 | Arms: 1  Target: DPA  Theory: SCT  Duration: ≤ 12 months | Control: Waitlist | Diet:   - *Energy* - Fruit intake - *Vegetable intake*   Physical activity:   - Light physical activity - *Moderate physical activity* - Vigorous physical activity - *Sedentary behaviour* |
| Levy 2012  Mexico | Design: C-RCT (School)  Setting: School  Age group: 6-12 (intv 78·6% = 10; control: 75·3% = 10)  Gender: mixed | 1020 | 997 | Arms: 1  Target: DPA  Theory: not reported  Duration: ≤ 12 months | Control: Usual care presumed as no details but school-based  intervention | Diet:   - *Energy*   Physical activity:   - *Total physical activity* - *Sedentary behaviour* |
| Li 2019  China | Design: C-RCT (School)  Setting: School* + Home  Age group: 6-12 (intv = 6·15, control = 6·14)  Gender: mixed | 1641 | 1581 | Arms: 1  Target: DPA  Theory: Behaviour change techniques, social marketing principles, MRC framework  Duration: ≤ 12 months | Control: Usual practice | Diet:   - *Fruit and vegetable intake*   Physical activity:   - *MVPA* - *Sedentary behaviour* |
| Liu 2019  China | Design: C-RCT (School)  Setting: School  Age group: 6-12 (9·0)  Gender: mixed | 1889 | 1839 | Arms: 1  Target: DPA  Theory: ANGELO framework, SCT  Duration: ≤ 12 months | Control: No intervention | Diet:   - *Fruit intake* - Vegetable intake - *Sugar-sweetened beverage intake*   Physical activity:   - *MVPA* |
| Llargues 2012  Spain | Design: C-RCT (School)  Setting: School  Age group: 6-12 (6·03)  Gender: mixed | 704 | 509 | Arms: 1  Target: DPA  Theory: Investigation, Vision, Action and Change (IVAC) Methodology  Duration: > 12 months | Control: Usual care presumed as no details but school-based  intervention | Diet:   - *Fruit and vegetable intake*   Physical activity:   - *physical activity outside school* |
| Lloyd 2018  England | Design: C-RCT (School)  Setting: School* + Home  Age group: 6-12 (intv = 9·8, control = 9·7)  Gender: mixed | 1324 | 1265 | Arms: 1  Target: DPA  Theory: Intervention mapping approach, behaviour change theories, HPSF  Duration: ≤ 12 months | Control: Usual practice | Physical activity:   - Accelerometer counts - Total physical activity - Light physical activity - *Moderate physical activity* - MVPA - *Sedentary behaviour* |
| Lubans 2011  Australia | Design: C-RCT (School)  Setting: School* + Home  Age group: 13-18 (intv = 14·4, control = 14·2)  Gender: boys only | 100 | 100 | Arms: 1  Target: physical activity  Theory: SCT  Duration: ≤ 12 months | Control: Waitlist | Diet:   - *Vegetable intake* - Fruit intake - *Sugar-sweetened beverage intake* - Water intake   Physical activity:   - *Total physical activity* |
| Luszczynska 2016  Poland | Design: RCT  Setting: School  Age group: 13-18 (16·35)  Gender: mixed | 702 | 506 | Arms: 2  Target: Diet  Theory: SCT, BCT, self efficacy or planning  Duration: ≤ 12 months | Control: Attention control. In the group component, participants were asked to read the materials and fill in the forms provided. Participants received a set of educational materials (including crosswords) about healthy nutrition, which focused on FV consumption | Diet:   - *Fruit and vegetable intake* |
| Luszczynska 2016b  Poland | Design: RCT  Setting: School  Age group: 13-18 (16·45)  Gender: mixed | 1217 | 1217 | Arms: 3  Target: physical activity  Theory: SCT, BCT, planning or self efficacy  Duration: ≤ 12 months | Control: Attention control - Education only. | Physical activity:   - *MVPA* |
| Lynch 2016  USA | Design: C-RCT (Classroom)  Setting: School  Age group: 6-12 (Median = 8)  Gender: mixed | 51 | 50 | Arms: 1  Target: DPA  Theory: not reported  Duration: ≤ 12 months | Control: Assume usual practice | Physical activity:   - *Accelerometer counts* |
| Madsen 2013  USA | Design: C-RCT (School)  Setting: School (ASP)  Age group: 6-12 (intv = 9·8, control = 9·8)  Gender: mixed | 156 | 150 | Arms: 1  Target: physical activity  Theory: not reported  Duration: ≤ 12 months | Control: No intervention presumed as no details provided | Physical activity:   - *MVPA* |
| Madsen 2015  USA | Design: C-RCT (School)  Setting: School* + Community  Age group: 6-12 (not reported)  Gender: mixed | 1079 | 676 | Arms: 1  Target: DPA  Theory: not reported  Duration: > 12 months | Control: Waitlist | Diet:   - Fruit intake - *Vegetable intake* - *Sugar-sweetened beverage intake*   Physical activity:   - *School day MVPA* - *School day sedentary behaviour* |
| Magnusson 2012  Iceland | Design: C-RCT (School)  Setting: School  Age group: 6-12 (intv = 7·3, control = 7·4)  Gender: mixed | 321 | 185 | Arms: 1  Target: DPA  Theory: not reported  Duration: > 12 months | Control: Usual practice + incentives | Diet:   - *Fruit and vegetable intake* |
| Marcus 2009  Sweden | Design: C-RCT (School)  Setting: School* and ASP)  Age group: 6-12 (intv = 7·4, control = 7·5)  Gender: mixed | 3135 | 2838 | Arms: 1  Target: DPA  Theory: not reported  Duration: > 12 months | Control: Normal curriculum | Diet:   - *Fruit and vegetable intake*   Physical activity:   - *Total physical activity* |
| Martinez-Vizcaino 2014  Spain | Design: C-RCT (School)  Setting: School (ASP)  Age group: 6-12 (intv = 9·4, control = 9·5)  Gender: mixed | 1592 | 912 | Arms: 1  Target: physical activity  Theory: SEM  Duration: ≤ 12 months | Control: Standard physical education curriculum(2 h/week of physical activity at low to moderate intensity) | Physical activity:   - *MVPA* - *Sedentary behaviour* |
| Mauriello 2010  USA | Design: C-RCT (School)  Setting: School  Age group: 13-18 (not reported)  Gender: mixed | 1800 | 1182 | Arms: 1  Target: DPA  Theory: TTM of Behaviour Change  Duration: ≤ 12 months | Control: No intervention | Diet:   - *Fruit and vegetable intake*   Physical activity:   - *Total physical activity* |
| Melnyk 2013  USA | Design: C-RCT (School)  Setting: School + Home  Age group: 13-18 (intv = 14·75, control = 14·74)  Gender: mixed | 807 | 627 | Arms: 1  Target: DPA  Theory: Cognitive theory  Duration: ≤ 12 months | Control: Attention control programme – safety and common health topics/issues | Physical activity:   - *Accelerometer counts* |
| Mihas 2010  Greece | Design: RCT  Setting: School  Age group: 13-18 (intv = 13·1, control = 13·3)  Gender: mixed | 213 | 191 | Arms: 1  Target: Diet  Theory: Social Learning theory  Duration: ≤ 12 months | Control: Usual care presumed as no details but school-based  intervention | Diet:   - *Energy* - *Fruit intake* - Vegetable intake |
| Muller 2019  South Africa | Design: C-RCT (School)  Setting: School  Age group: 6-12 (intv 1 = 10.0, intv 2 = 10.1, control = 9·9)  Gender: mixed | 1009 | 519 | Arms: 3  Target: physical activity  Theory: not reported  Duration: ≤ 12 months | Control: Usual practice | Physical activity:   - *Mean physical activity* |
| Neumark-Sztainer 2003  USA | Design: C-RCT (School)  Setting: School* + Home  Age group: 13-18 (intv = 14·9, control = 15·8)  Gender: Girls only | 201 | 190 | Arms: 1  Target: DPA  Theory: SCT  Duration: ≤ 12 months | Control: Regular physical education class and minimal intervention  (written materials on healthy eating  and physical activity at baseline) | Diet:   - *Fruit and vegetable intake* - *Soft drink intake*   Physical activity:   - *Total physical activity* - *Sedentary behaviour* |
| Neumark-Sztainer 2010  USA | Design: C-RCT (School)  Setting: School* + Home  Age group: 13-18 (15·8)  Gender: Girls only | 356 | 336 | Arms: 1  Target: DPA  Theory: SCT, Stages of Change  Duration: > 12 months | Control: All-girls PE class during the first semester then usual PE | Diet:   - *Fruit and vegetable intake* - *Sugar-sweetened beverage intake*   Physical activity:   - *Total physical activity* - MVPA - *Sedentary behaviour* |
| Nyberg 2015  Sweden | Design: C-RCT (Classroom)  Setting: School* + Home  Age group: 6-12 (intv = 6·2, control = 6·2)  Gender: mixed | 243 | 239 | Arms: 1  Target: DPA  Theory: SCT  Duration: ≤ 12 months | Control: Waitlist | Diet:   - *Fruit intake* - Vegetable intake - *Fruit juice intake* - Soft drink intake - Flavoured milk intake   Physical activity:   - Total physical activity - *MVPA* - *Sedentary behaviour* |
| Nyberg 2016  Sweden | Design: C-RCT (Classroom)  Setting: Preschool*+ Home  Age group: 6-12 (6.3)  Gender: mixed | 378 | 332 | Arms: 1  Target: DPA  Theory: SCT  Duration: ≤ 12 months | Control: Waitlist | Diet:   - *Fruit intake* - Vegetable intake - *Milk intake* - Fruit juice intake - *Soft drink intake* - Flavoured milk intake   Physical activity:   - Total physical activity - *MVPA* - *Sedentary behaviour* |
| Pate 2005  USA | Design: C-RCT (School)  Setting: School + Home Ccommunity  Age group: 13-18 (13·6)  Gender: Girls only | 1604 | 1539 | Arms: 1  Target: physical activity  Theory: SEM drawn from SCT  Duration: ≤ 12 months | Control: Usual care | Physical activity:   - MVPA - *Vigorous physical activity* |
| Peralta 2009  Australia | Design: RCT  Setting: School* + Home  Age group: 13-18 (12·5)  Gender: Boys only | 33 | 33 | Arms: 1  Target: DPA  Theory: SCT  Duration: ≤ 12 months | Control: Usual care. physical activity curriculum sessions | Diet:   - *Fruit intake* - *Sugar-sweetened beverage intake*   Physical activity:   - *Moderate physical activity* - Vigorous physical activity - MVPA - Accelerometer counts |
| Pfeiffer 2019  USA | Design: C-RCT (School)  Setting: School (ASP)  Age group: 6-12 (12·05)  Gender: Girls only | 1519 | 1519 | Arms: 1  Target: physical activity  Theory: Health promotion model, self-determination theory  Duration: ≤ 12 months | Control: Usual practice | Physical activity:   - Light physical activity - *MVPA* - *Sedentary behaviour* |
| Ramirez-Rivera 2021  Mexico | Design: RCT  Setting: School* + Home  Age group: 6-12 (10·2)  Gender: mixed | 41 | 41 | Arms: 1  Target: DPA  Theory: not reported  Duration: ≤ 12 months | Control: General nutrition recommendations | Physical activity:   - *Total physical activity* - *Sedentary behaviour* |
| Reed 2008  Canada | Design: C-RCT (School)  Setting: School* + Home  Age group: 6-12 (not reported)  Gender: mixed | 268 | 237 | Arms: 1  Target: physical activity  Theory: SEM  Duration: ≤ 12 months | Control: Usual care | Physical activity:   - *Total physical activity* - MVPA |
| Robbins 2006  USA | Design: C-RCT (Grade)  Setting: School* + Home  Age group: 6-12 (intv grade 6 = 11·45, grade 7 = 12·37, grade 8 = 13·00; control grade 6 = 11·25, grade 7 = 12·27, grade 8 = 13·44)  Gender: Girls only | 77 | 77 | Arms: 1  Target: physical activity  Theory: Health Promotion Model and TTM  Duration: ≤ 12 months | Attention control: Handout listing the physical activity recommendations | Physical activity:   - Moderate physical activity - MVPA - *Vigorous physical activity* |
| Rosario 2012  Portugal | Design: C-RCT (School)  Setting: School  Age group: 6-12 (8·3)  Gender: mixed | 464 | 294 | Arms: 1  Target: Diet  Theory: Health Promotion Model and SCT  Duration: ≤ 12 months | Control: Usual care presumed as no details but school-based  intervention | Diet:   - *Energy* - *Fruit and vegetable intake* - *Fruit juice intake*   Physical activity   - *Low, moderate and vigorous activity* - *Sedentary behaviour* |
| Safdie 2013  Mexico | Design: C-RCT (School)  Setting: School  Age group: 6-12 (intv plus = 9·7, intv basic = 9·7, control = 9·8)  Gender: mixed | 886 | 830 | Arms: 2  Target: DPA  Theory: Ecological principles, Theory of Planned Behaviour, SCT , Health Belief Model  Duration: > 12 months | Control: No changes were made to existing nutrition or physical activity practices | Physical activity:   - *Accelerometer counts* |
| Sahota 2001  United Kingdom | Design: C-RCT - crossover (School)  Setting: School  Age group: 6-12 (intv = 8·36, control = 8·42)  Gender: mixed | 613 | 595 | Arms: 1  Target: physical activity  Theory: multi-component health promotion programme, based on the Health Promoting Schools concept  Duration: ≤ 12 months | Control: Usual care presumed as no details but school-based  intervention | Diet:   - Fruit intake - *Vegetable intake*   Physical activity:   - *Total physical activity* - *Sedentary behaviour* |
| Sahota 2019  England | Design: C-RCT (School)  Setting: School  Age group: 6-12 (year 2 intv = 6·2, control = 6·3; year 4 = 8·3; overall 7·2)  Gender: mixed | 358 | 311 | Arms: 1  Target: DPA  Theory: Behaviour Theory, BCW  Duration: > 12 months | Control: Usual practice | Diet:   - *Fruit intake* - Vegetable intake - *Water intake* - Diet soft drink - Sugar-sweetened soft drinks |
| Sallis 1993  USA | Design: C-RCT (School)  Setting: School  Age group: 6-12 (9·25)  Gender: mixed | 745 | 549 | Arms: 2  Target: physical activity  Theory: Behaviour Change and self-management  Duration: > 12 months | Control: Usual care PE | Physical activity:   - Vigorous activity - *MVPA* - *Sitting time* - Lying time |
| Salmon 2008  Australia | Design: C-RCT (Classroom)  Setting: School  Age group: 6-12 (10·7)  Gender: mixed | 295 | 268 | Arms: 3  Target: physical activity  Theory: SCT and Behavioural Choice theory  Duration: ≤ 12 months | Control: Usual care curriculum | Physical activity:   - Accelerometer counts - Moderate physical activity - *Vigorous physical activity* |
| Santos 2014  Canada | Design: C-RCT (School)  Setting: School  Age group: 6-12 (intv = 9·3, control = 8·8)  Gender: mixed | 687 | 647 | Arms: 1  Target: DPA  Theory: not reported  Duration: ≤ 12 months | Control: Usual care regular curriculum | Physical activity:   - *Accelerometer counts* |
| Sgambato 2019  Brazil | Design: C-RCT (School)  Setting: School* + Home  Age group: 6-12 (not reported)  Gender: mixed | 2743 | 2276 | Arms: 1  Target: DPA  Theory: not reported  Duration: ≤ 12 months | Control: Assume usual practice | Diet:   - *Fruit intake* - Vegetable intake - *Water intake* - Soft drink intake - Fruit-based drink intake   Physical activity:   - *Total physical activity* |
| Sichieri 2008  Brazil | Design: C-RCT (School)  Setting: School  Age group: 6-12 (10·9)  Gender: mixed | 1134 | 927 | Arms: 1  Target: Diet  Theory: not reported  Duration: ≤ 12 months | Attention control: 2 x 1-h general sessions on health issues and printed general advices regarding healthy diets | Diet:   - *Soft drink intake* - Fruit juice intake |
| Siegrist 2013  Germany | Design: C-RCT (School)  Setting: School* + Home  Age group: 6-12 (8·4)  Gender: mixed | 826 | 724 | Arms: 1  Target: DPA  Theory: not reported  Duration: ≤ 12 months | Control: Usual care | Physical activity:   - *Total physical activity* |
| Siegrist 2018  Germany | Design: C-RCT (School)  Setting: School* + Home  Age group: 6-12 (11·1)  Gender: mixed | 620 | 434 | Arms: 1  Target: DPA  Theory: SCT  Duration: > 12 months | Control: Usual practice - normal PE program | Diet:   - *Soft drink intake*   Physical activity:   - *Total physical activity* |
| Simon 2008  France | Design: C-RCT (School)  Setting: School* + ASP  Age group: 6-12 (intv = 11·7, control = 11·6)  Gender: mixed | 954 | 954 | Arms: 1  Target: physical activity  Theory: Behaviour Change and SEM  Duration: > 12 months | Control: Usual care school curriculum | Physical activity:   - *Leisure time physical activity* |
| Singh 2009  The Netherlands | Design: C-RCT (School)  Setting: School  Age group: 13-18 (intv boys = 12·8, girls = 12·6; control boys = 12·9, girls = 12·7)  Gender: mixed | 1108 | 1108 | Arms: 1  Target: DPA  Theory: Intervention mapping protocol, Behaviour Change and Environmental frameworks  Duration: ≤ 12 months | Control: Usual care regular curriculum | Diet:   - *Sugar-sweetened beverage intake and fruit juice intake* |
| Smith 2014  Australia | Design: C-RCT (School)  Setting: School* + Home  Age group: 13-18 (12·7)  Gender: Boys only | 361 | 361 | Arms: 1  Target: physical activity  Theory: Self-determination theory and SCT  Duration: ≤ 12 months | Control: Waitlist and usual practice (i.e. regularly scheduled  school sports and PE) | Diet:   - *Sugar-sweetened beverage intake*   Physical activity:   - Total physical activity - *MVPA* |
| Story 2003a  USA | Design: RCT  Setting: School* + Home  Age group: 6-12 (intv = 9·4, control = 9·1)  Gender: Girls only | 53 | 53 | Arms: 1  Target: DPA  Theory: SCT, youth development, and resiliency based approach  Duration: ≤ 12 months | Control: “active placebo,” non-nutrition/physical activity condition, promoting self-esteem and cultural enot reportedichment | Diet:   - *Energy* - *Fruit and vegetable intake* - Sugar-sweetened beverage intake - *Water intake*   Physical activity:   - *Accelerometer counts* |
| Telford 2012  Australia | Design: C-RCT (Schools)  Setting: School  Age group: 6-12 (not reported)  Gender: mixed | Unclear | 620 | Arms: 1  Target: physical activity  Theory: not reported  Duration: > 12 months | Control: Usual care, common practice PE | Physical activity:   - *Accelerometer counts* |
| TenHoor 2018  The Netherlands | Design: C-RCT (School)  Setting: School  Age group: 13-18 (12·97)  Gender: mixed | 695 | 293 | Arms: 1  Target: physical activity  Theory: not reported  Duration: ≤ 12 months | Control: Usual curriculum | Physical activity:   - *Accelerometer counts* - Light physical activity - MVPA |
| Viggiano 2018  Italy | Design: C-RCT (School)  Setting: School  Age group: 6-12 (not reported)  Gender: mixed | 1313 | 1007 | Arms: 1  Target: DPA  Theory: not reported  Duration: ≤ 12 months | Control: No intervention | Physical activity:   - physical activity frequency - *physical activity duration* |
| Wang 2018  China | Design: C-RCT (School)  Setting: School* + Community + Home  Age group: 6-12 (10·5)  Gender: mixed | 10091 | 9858 | Arms: 1  Target: DPA  Theory: not reported  Duration: ≤ 12 months | Control: No intervention | Physical activity:   - *Mean moderate physical activity* |
| Warren 2003  England | Design: RCT  Setting: School* + Home  Age group: 6-12 (6·1)  Gender: mixed | 218 | 172 | Arms: 3  Target: Diet, physical activity, DPA  Theory: Social Learning theory  Duration: > 12 months | Control: Educational programme about food in a ‘non-nutrition’ = Be Smart  sense | Diet:   - *Fruit intake* - Vegetable intake |
| Waters 2017  Australia | Design: C-RCT (School)  Setting: School* + Community + Home  Age group: 6-12 (not reported)  Gender: mixed | 3222 | 2743 | Arms: 1  Target: DPA  Theory: Health Promoting Schools Framework (based on health promotion theory and consistent with a socio-environmental theoretical framework) and International Obesity Task Force ‘10 guiding principles for obesity prevention'  Duration: > 12 months | Control: Usual practice | Diet:   - Fruit intake - *Vegetable intake* - *Soft drink intake* - Fruit juice/cordial intake   Physical activity:   - *Active games at lunch time* |
| Wendel 2016  USA | Design: C-RCT (Classroom)  Setting: School  Age group: 6-12 (8·8)  Gender: mixed | 343 | 111 | Arms: 3  Target: physical activity  Theory: not reported  Duration: > 12 months | Control: No intervention | Physical activity:   - *Accelerometer counts* |
| White 2019  USA | Design: RCT  Setting: Community* + Home  Age group: 6-12 (9·35)  Gender: mixed | 228 | 125 | Arms: 1  Target: DPA  Theory: SCT, experiential 4-H learning model  Duration: > 12 months | Control: No intervention | Physical activity:   - Light physical activity - Moderate physical activity - *Vigorous physical activity* - MVPA - *Sedentary behaviour* |
| Wilksch 2015  Australia | Design: C-RCT (Classroom)  Setting: School  Age group: 13-18 (13·21)  Gender: mixed | 820 | 820 | Arms: 1  Target: DPA  Theory: not reported  Duration: ≤ 12 months | Control: Usual school class | Physical activity:   - *Total physical activity* |
| Williamson 2012  USA | Design: C-RCT (School)  Setting: School* + Home  Age group: 6-12 (Primary Prevention = 10·5, Secondary Prevention = 10·5, control = 10·6)  Gender: mixed | 2060 | 1697 | Arms: 2  Target: DPA  Theory: SLT  Duration: > 12 months | Control: No intervention | Diet:   - *Energy*   Physical activity:   - *Total physical activity* - *Sedentary behaviour* |
| Xu 2015  China | Design: C-RCT (School)  Setting: School* + Home  Age group: 6-12 (10·2)  Gender: mixed | 1182 | 1108 | Arms: 1  Target: DPA  Theory: not reported  Duration: ≤ 12 months | Control: Usual practice | Diet:   - *Vegetable intake* - *Soft drink intake*   Physical activity:   - Running - *Walking* - Ball playing |
| Xu 2017  China | Design: C-RCT (School)  Setting: School* + Home  Age group: 6-12 (9·2)  Gender: mixed | 9867 | 8573 | Arms: 3  Target: Diet, physical activity, DPA  Theory: not reported  Duration: ≤ 12 months | Control: no intervention | Diet:   - *Energy* - Fruit intake - *Vegetable intake* |
| Zhou 2019  China | Design: C-RCT (School)  Setting: School* (some groups ASP) + Home  Age group: 13-18 (12·66)  Gender: mixed | 758 | 681 | Arms: 3  Target: DPA  Theory: SEM, CMT  Duration: ≤ 12 months | Control: Usual care | Physical activity:   - Light physical activity - Moderate physical activity - Vigorous physical activity - *MVPA* - *Sedentary behaviour* |
| Zota 2016  Greece | Design: C-RCT (School)  Setting: School* + Home  Age group: 6-12; 13-18 (not reported)  Gender: mixed | 21261 | 3627 | Arms: 1  Target: Diet  Theory: not reported  Duration: ≤ 12 months | Attention control: environmental intervention (received a healthy daily meal) | Diet:   - *Fruit intake* - Vegetable intake |

*Majority setting - where the intervention mainly took place if there was more than one setting

ANGELO = ANalysis Grid for Environments Linked to Obesity, ASE = Attitude, social influence and self-efficacy model, ASP = after school programs, BCT = Behaviour Change Theories, BCW = Behaviour Change Wheel, CBT = cognitive behavioural therapy, CDC = Centres for Disease Control, COM-B = capability, opportunity, motivation – behaviour, CMT = competence motivation theory, C-RCT = cluster randomised controlled trial, DPA = diet and physical activity, HPSF = Health Promoting Schools Framework, IMB model = information-motivation-behavioural skills model, Intv = intervention, MRC = Medical Research Council, MVPA = moderate-vigorous physical activity, PE = physical education, SEM = Social Ecological Model, SCT = Social Cognitive Theory, SLT = Social learning theory, TDF = theoretical domains framework, TPB = Theory of planned behaviour, TTM = Transtheoretical model (Stages of Change), USA = United States of America
