# Supplementary material for: The effectiveness of school-based obesity prevention interventions on the health behaviours of children aged 6–18 years: A secondary data analysis of a systematic review
Source: Prev Med Rep. 2025 Mar 31;53:103053. doi: 10.1016/j.pmedr.2025.103053 (PMC11999465; doi:10.1016/j.pmedr.2025.103053)
Supplement: Supplementary file 2 — Supplementary material 2 [file mmc2.docx]

**Appendix b**

**Table S2.** Risk of bias of included studies assessing school obesity prevention interventions in children from 1990-2023

| Study Identification | Design | Intervention type | Outcome | Random sequence (selection bias) | Allocation concealment (selection bias) | Blinding (performance and detection bias) | Incomplete outcome data (attrition bias) | Selective outcome reporting  (reporting bias) | Other overall | Overall risk of bias |
| --- | --- | --- | --- | --- | --- | --- | --- | --- | --- | --- |
| Adab 2018 | C-RCT | DPA | Energy intake | Low | Low | High | High | High | High | High |
|  |  |  | Fruit and vegetable intake | Low | Low | High | High | Low | High | High |
|  |  |  | MVPA | Low | Low | High | High | High | High | High |
|  |  |  | Sedentary behaviour | Low | Low | High | High | High | High | High |
| Amaro 2006 | C-RCT | D | Vegetable intake | Unclear | Unclear | High | Low | Unclear | Low | Unclear |
|  |  |  | Total PA | Unclear | Unclear | High | Low | Unclear | Low | Unclear |
| Andrade 2014 | C-RCT | DPA | Fruit and vegetable intake | Low | Low | High | High | High | Low | High |
|  |  |  | Sedentary behaviour | Low | Low | High | High | Low | Low | High |
|  |  |  | MVPA | Low | Low | High | High | High | Low | High |
| Arlinghaus 2021 | RCT | PA | MVPA | Low | Unclear | High | High | Low | Low | High |
| Barbeau 2007 | RCT | PA | MVPA | Unclear | Unclear | High | High | Unclear | Low | Unclear |
| Bohnert 2013 | RCT | DPA | Vegetable intake | High | High | High | High | Unclear | Low | High |
|  |  |  | Total PA | High | High | High | High | Unclear | Low | High |
| Bonsergent 2013 | C-RCT | DPA | Total PA | Low | Low | High | High | Low | Low | High |
|  |  |  | Sedentary behaviour | Low | Low | High | High | Low | Low | High |
| Brito Beck da Silva 2019 | C-RCT | DPA | Fruit intake | Unclear | Unclear | High | High | High | Unclear | High |
|  |  |  | Soft drink intake | Unclear | Unclear | High | High | High | Unclear | High |
| Caballero 2003 | C-RCT | DPA | Energy intake | Low | Unclear | Unclear | Low | High | Low | Unclear |
|  |  |  | Total PA | Low | Unclear | Unclear | High | Low | Low | Unclear |
| Christiansen 2013 | C-RCT | PA | Sedentary behaviour | Unclear | Unclear | Unclear | High | Low | Low | High |
|  |  |  | MVPA | Unclear | Unclear | Unclear | High | Low | Low | High |
| Clemes 2020 | C-RCT | PA | Sedentary behaviour | Low | Unclear | Low | Low | Low | Low | Unclear |
|  |  |  | MVPA | Low | Unclear | Low | Low | Low | Low | Unclear |
| Cunha 2013 | C-RCT | D | Fruit intake | Unclear | Low | High | Low | High | Unclear | Unclear |
|  |  |  | Juice intake |  | Low | High | Low | Low | Unclear | Unclear |
| Damsgaard 2014 | C-RCT | D | Energy intake | Low | High | High | Low | Low | Unclear | High |
|  |  |  | Vegetable intake | Low | High | High | Low | Low | Unclear | High |
| Davis 2021 | C-RCT | D | Energy intake | Low | Unclear | High | High | High | High | High |
|  |  |  | Vegetable intake | Low | Unclear | High | Low | High | High | High |
|  |  |  | SSB intake | Low | Unclear | High | Low | High | High | High |
| Dewar 2013 | C-RCT | DPA | Total PA | Unclear | Low | Unclear | Low | High | Low | High |
|  |  |  | Sedentary behaviour | Unclear | Low | Unclear | High | Low | Low | High |
|  |  |  | Energy intake | Unclear | Low | High | High | High | Low | High |
| Donnelly 2009 | C-RCT | PA | Total PA | Unclear | Unclear | Unclear | Low | Unclear | Low | High |
| Drummy 2016 | C-RCT | PA | MVPA | Unclear | Unclear | Unclear | Unclear | Unclear | High | High |
| Duncan 2019 | C-RCT | DPA | Fruit intake | Low | Unclear | Unclear | Low | Low | High | High |
|  |  |  | SSB intake | Low | Unclear | Unclear | Low | Low | High | High |
|  |  |  | Total PA | Low | Unclear | Unclear | Low | Low | High | High |
| Dunker 2018 | C-RCT | DPA | Fruit intake | Unclear | Unclear | High | Low | High | Unclear | Unclear |
|  |  |  | Artificial juice intake | Unclear | Unclear | High | Low | High | Unclear | Unclear |
|  |  |  | Total PA | Unclear | Unclear | High | High | High | Unclear | Unclear |
|  |  |  | Sedentary behaviour | Unclear | Unclear | High | High | High | Unclear | Unclear |
| Ezendam 2012 | C-RCT | DPA | Fruit intake | Low | Low | High | Low | Low | Low | High |
|  |  |  | SSB intake | Low | Low | High | Low | Low | Low | High |
|  |  |  | Total PA | Low | Low | Unclear | Low | Low | Low | High |
| Fairclough 2013 | C-RCT | DPA | Vegetable intake | Low | High | High | Low | High | Low | High |
|  |  |  | Light PA | Low | High | Unclear | Low | High | Low | High |
|  |  |  | Sedentary behaviour | Low | High | Unclear | Low | High | Low | High |
| Farmer 2017 | C-RCT | PA | Total PA | Low | Unclear | Unclear | Low | Unclear | Unclear | High |
| Foster 2008 | C-RCT | DPA | Energy intake | Unclear | Unclear | High | High | Unclear | Low | Unclear |
|  |  |  | Fruit and vegetable intake | Unclear | Unclear | High | High | Unclear | Low | Unclear |
|  |  |  | Total PA | Unclear | Unclear | High | High | Unclear | Low | Unclear |
|  |  |  | Sedentary behaviour | Unclear | Unclear | High | High | Unclear | Low | Unclear |
| Gentile 2009 | C-RCT | DPA | Fruit and vegetable intake | Unclear | Unclear | Unclear | Unclear | Low | Low | High |
|  |  |  | Total PA | Unclear | Unclear | Unclear | Unclear | Low | Low | High |
| Gortmaker 1999a | C-RCT | DPA | Energy intake | Low | Low | High | Unclear | Unclear | Low | High |
|  |  |  | Fruit and vegetable intake | Low | Low | High | Unclear | Unclear | Low | High |
|  |  |  | MVPA | Low | Low | High | Unclear | Unclear | Low | High |
| Grydeland 2014 | C-RCT | DPA | Vegetable intake | Unclear | Unclear | High | Low | Low | Unclear | Unclear |
|  |  |  | Soft drink intake | Unclear | Unclear | High | Low | Low | Unclear | Unclear |
|  |  |  | Sedentary behaviour | Unclear | Unclear | High | High | Low | Unclear | Unclear |
|  |  |  | MVPA | Unclear | Unclear | High | High | Low | Unclear | Unclear |
| Habib-Mourad 2014 | C-RCT | DPA | Fruit intake | Low | High | High | Low | Unclear | Low | High |
|  |  |  | Soft drink intake | Low | High | High | Low | Unclear | Low | High |
|  |  |  | Recess PA | Low | High | High | Low | Unclear | Low | High |
| Habib-Mourad 2020 | C-RCT | DPA | Fruit intake | Unclear | Unclear | High | High | High | High | High |
|  |  |  | Soft drink intake | Unclear | Unclear | High | High | High | High | High |
|  |  |  | Exercise after school | Unclear | Unclear | High | High | High | High | High |
| Haerens 2006 | C-RCT | DPA | Fruit intake | Unclear | Unclear | High | Low | Unclear | Low | High |
|  |  |  | Water intake | Unclear | Unclear | High | Low | Unclear | Low | High |
|  |  |  | MVPA | Unclear | Unclear | Unclear | Low | Unclear | Low | High |
|  |  |  | Sedentary behaviour | Unclear | Unclear | Unclear | Low | Unclear | Low | High |
| Harrington 2018 | C-RCT | PA | MVPA | Low | Low | Unclear | Low | Low | High | High |
|  |  |  | Sedentary behaviour | Low | Low | Unclear | Low | Low | High | High |
| HEALTHY Study Gp 2010 | C-RCT | DPA | Energy intake | Low | Low | High | Low | High | Unclear | High |
|  |  |  | Fruit and vegetable intake | Low | Low | High | Low | High | Unclear | High |
|  |  |  | Water intake | Low | Low | High | Low | High | Unclear | High |
| Herscovici 2013 | C-RCT | DPA | Vegetable intake | Unclear | Unclear | High | Low | Unclear | Low | Unclear |
|  |  |  | Juice intake | Unclear | Unclear | High | Low | Unclear | Low | Unclear |
| Hollis 2016 | C-RCT | PA | Moderate PA | Low | Low | Unclear | High | High | Low | Unclear |
| Howe 2011 | RCT | PA | MVPA | High | Unclear | High | Low | Unclear | Low | High |
| Ickovics 2019 | C-RCT | D, PA, DPA | Total PA | Low | Unclear | High | Low | High | High | High |
| James 2004 | C-RCT | D | SSB intake | Low | Low | High | Low | Unclear | Low | Unclear |
| Kennedy 2018 | C-RCT | PA | MVPA | Low | Unclear | Unclear | High | Low | Unclear | Unclear |
| Kipping 2014 | C-RCT | DPA | Fruit and vegetable intake | Low | Low | High | Unclear | Low | Low | Low |
|  |  |  | MVPA | Low | Low | Unclear | Low | Low | Low | Low |
|  |  |  | Sedentary behaviour | Low | Low | Unclear | Low | Low | Low | Low |
| Kobel 2017 | C-RCT | DPA | Fruit and vegetable intake | Unclear | Unclear | Unclear | High | High | High | High |
|  |  |  | Soft drink intake | Unclear | Unclear | Unclear | Low | High | High | High |
|  |  |  | MVPA | Unclear | Unclear | Unclear | Low | High | High | High |
| Kocken 2016 | C-RCT | DPA | Energy intake | Unclear | Unclear | High | Low | Unclear | High | High |
|  |  |  | Vegetable intake | Unclear | Unclear | High | Low | Unclear | High | High |
|  |  |  | Soft drink intake | Unclear | Unclear | High | Low | Unclear | High | High |
|  |  |  | Sedentary behaviour | Unclear | Unclear | Unclear | High | Unclear | High | High |
|  |  |  | MVPA | Unclear | Unclear | Unclear | High | Unclear | High | High |
| Kriemler 2010 | C-RCT | PA | Total PA | Low | Low | Unclear | High | Low | Low | High |
| Kubik 2021 | RCT | DPA | Energy intake | Low | Unclear | High | Low | High | Low | Unclear |
|  |  |  | Fruit intake | Low | Unclear | High | Low | High | Low | Unclear |
|  |  |  | SSB intake | Low | Unclear | High | Low | High | Low | Unclear |
|  |  |  | Light PA | Low | Unclear | High | High | Low | Low | Unclear |
|  |  |  | Sedentary behaviour | Low | Unclear | High | High | Low | Low | Unclear |
| Lana 2014 | RCT | D, PA | Smoking | Low | Low | High | High | Low | Low | High |
|  |  |  | Alcohol | Low | Low | High | High | Low | Low | High |
|  |  |  | Fruit intake | Low | Low | High | High | Low | Low | High |
|  |  |  | Sedentary behaviour | Low | Low | High | High | Low | Low | High |
| Leme 2016 | C-RCT | DPA | Energy intake | Low | Low | High | High | High | High | High |
|  |  |  | Vegetable intake | Low | Low | High | High | High | High | High |
|  |  |  | Moderate PA | Low | Low | High | High | Low | High | High |
|  |  |  | Sedentary behaviour | Low | Low | High | High | Low | High | High |
| Levy 2012 | C-RCT | DPA | Energy intake | Unclear | Unclear | High | Low | Unclear | Low | Unclear |
|  |  |  | Total PA | Unclear | Unclear | High | Low | Unclear | Low | Unclear |
|  |  |  | Sedentary behaviour | Unclear | Unclear | High | Low | Unclear | Low | Unclear |
| Li 2019 | C-RCT | DPA | Fruit and vegetable intake | Low | Unclear | High | Low | High | Low | Unclear |
|  |  |  | MVPA | Low | Unclear | Unclear | Low | Low | Low | Unclear |
|  |  |  | Sedentary behaviour | Low | Unclear | Unclear | Low | Low | Low | Unclear |
| Liu 2019 | C-RCT | DPA | Fruit intake | Low | Unclear | High | Unclear | High | Unclear | Unclear |
|  |  |  | SSB intake | Low | Unclear | High | Unclear | High | Unclear | Unclear |
|  |  |  | Total PA | Low | Unclear | High | Low | High | Unclear | Unclear |
| Llargues 2012 | C-RCT | DPA | Sedentary behaviour | Unclear | Unclear | High | Low | High | Low | High |
|  |  |  | Total PA | Unclear | Unclear | High | Low | High | Low | High |
|  |  |  | Vegetable intake | Unclear | Unclear | High | Low | High | Low | High |
| Lloyd 2018 | C-RCT | DPA | Moderate PA | Low | Low | High | Low | Low | Low | Low |
|  |  |  | Sedentary behaviour | Low | Low | High | Low | Low | Low | Low |
| Lubans 2011 | C-RCT | PA | Vegetable intake | Unclear | Unclear | High | Low | High | Low | Unclear |
|  |  |  | SSB intake | Unclear | Unclear | High | Low | High | Low | Unclear |
|  |  |  | Total PA | Unclear | Unclear | High | Low | High | Low | Unclear |
| Luszczynska 2016 | RCT | D | Fruit and vegetable intake | Low | High | Low | High | Unclear | Unclear | High |
| Luszczynska 2016b | RCT | PA | Total PA | Low | High | Low | Low | Unclear | Unclear | High |
| Lynch 2016 | C-RCT | DPA | Total PA | Low | Unclear | Unclear | High | Unclear | High | High |
| Madsen 2013 | C-RCT | PA | Total PA | Unclear | Unclear | High | Low | High | Low | Unclear |
| Madsen 2015 | C-RCT | DPA | Vegetable intake | Unclear | Unclear | Unclear | Low | Low | Unclear | High |
|  |  |  | SSB intake | Unclear | Unclear | High | High | Low | Unclear | High |
|  |  |  | MVPA | Unclear | Unclear | Unclear | High | Low | Unclear | High |
|  |  |  | Sedentary behaviour | Unclear | Unclear | Unclear | High | Low | Unclear | High |
| Magnusson 2012 | C-RCT | DPA | Fruit and vegetable intake | Unclear | Unclear | Unclear | Unclear | Unclear | Low | High |
| Marcus 2009 | C-RCT | DPA | Total PA | Unclear | Unclear | Unclear | Low | Unclear | Low | Unclear |
| Martinez-Vizcaino 2014 | C-RCT | PA | MVPA | Low | Low | High | Low | High | Low | Low |
|  |  |  | Sedentary behaviour | Low | Low | High | Low | High | Low | Low |
| Mauriello 2010 | C-RCT | DPA | Fruit and vegetable intake | Unclear | Unclear | High | High | Unclear | Low | High |
|  |  |  | Total PA | Unclear | Unclear | High | High | Unclear | Low | High |
| Melnyk 2013 | C-RCT | DPA | Total PA | Unclear | Unclear | Low | Low | Low | Low | High |
|  |  |  | Alcohol | Unclear | Unclear | Low | Low | High | Low | High |
| Mihas 2010 | RCT | D | Energy intake | Low | Unclear | High | Low | Unclear | High | High |
|  |  |  | Fruit intake | Low | Unclear | High | Low | Unclear | High | High |
| Muller 2019 | C-RCT | PA | Total PA | Low | Unclear | High | High | Low | Low | High |
| Neumark-Sztainer 2003 | C-RCT | DPA | Fruit and vegetable intake | Unclear | High | High | Low | Unclear | Low | High |
|  |  |  | SSB intake | Unclear | High | High | Low | Unclear | Low | High |
|  |  |  | Total PA | Unclear | High | High | Low | Unclear | Low | High |
|  |  |  | Sedentary behaviour | Unclear | High | High | Low | Unclear | Low | High |
| Neumark-Sztainer 2010 | C-RCT | DPA | Fruit and vegetable intake | Unclear | Unclear | High | Low | Unclear | Low | High |
|  |  |  | SSB intake | Unclear | Unclear | High | Low | Unclear | Low | High |
|  |  |  | Total PA | Unclear | Unclear | High | Low | Unclear | Low | High |
|  |  |  | Sedentary behaviour | Unclear | Unclear | High | Low | Unclear | Low | High |
| Nyberg 2015 | C-RCT | DPA | Fruit intake | Unclear | Unclear | Unclear | Low | Low | Low | Unclear |
|  |  |  | Soft drink intake | Unclear | Unclear | Unclear | High | High | Low | Unclear |
|  |  |  | MVPA | Unclear | Unclear | Unclear | Low | Low | Low | Unclear |
|  |  |  | Sedentary behaviour | Unclear | Unclear | Unclear | Low | Low | Low | Unclear |
| Nyberg 2016 | C-RCT | DPA | Fruit intake | Low | Unclear | Unclear | High | Low | Unclear | Unclear |
|  |  |  | Soft drink intake | Low | Unclear | Unclear | High | High | Unclear | Unclear |
|  |  |  | MVPA | Low | Unclear | Unclear | High | Low | Unclear | Unclear |
|  |  |  | Sedentary behaviour | Low | Unclear | Unclear | High | Low | Unclear | Unclear |
| Pate 2005 | C-RCT | PA | Vigorous PA | Unclear | Unclear | High | High | Unclear | Low | High |
| Peralta 2009 | RCT | DPA | Fruit intake | Low | Unclear | High | Low | Unclear | Low | Unclear |
|  |  |  | Moderate PA | Low | Unclear | Unclear | Low | Unclear | Low | Unclear |
|  |  |  | SSB intake | Low | Unclear | High | Low | Unclear | Low | Unclear |
| Pfeiffer 2019 | C-RCT | PA | MVPA | Low | Unclear | Unclear | High | Low | Unclear | Unclear |
|  |  |  | Sedentary behaviour | Low | Unclear | Unclear | High | High | Unclear | Unclear |
| Ramirez-Rivera 2021 | RCT | DPA | Total PA | Low | Low | High | Low | Low | Unclear | Unclear |
|  |  |  | Sedentary behaviour | Low | Low | High | Low | Low | Unclear | Unclear |
| Reed 2008 | C-RCT | PA | Total PA | Unclear | Low | Unclear | Unclear | High | Low | Unclear |
| Robbins 2006 | C-RCT | PA | Vigorous PA | Low | Low | High | Low | Unclear | Low | Unclear |
| Rosario 2012 | C-RCT | D | Energy intake | Low | Low | Low | High | Unclear | Low | High |
|  |  |  | Fruit and vegetable intake | Low | Low | Low | High | Unclear | Low | High |
|  |  |  | Total PA | Low | Low | Unclear | High | High | Low | High |
|  |  |  | Fruit juice intake | Low | Low | Low | High | Unclear | Low | High |
|  |  |  | Sedentary behaviour | Low | Low | Unclear | High | High | Low | High |
| Safdie 2013 | C-RCT | DPA | Total PA | Unclear | Unclear | High | Low | Unclear | Low | High |
| Sahota 2001 | C-RCT | PA | Vegetable intake | Low | Low | High | Low | Unclear | Low | Unclear |
|  |  |  | Total PA | Low | Low | High | Low | Unclear | Low | Unclear |
|  |  |  | Sedentary behaviour | Low | Low | High | Low | Unclear | Low | Unclear |
| Sahota 2019 | C-RCT | DPA | Fruit intake | Low | Unclear | High | Low | Low | High | High |
|  |  |  | Water intake | Low | Unclear | High | Low | Low | High | High |
| Sallis 1993 | C-RCT | PA | MVPA | High | Unclear | Unclear | Unclear | Unclear | Unclear | High |
|  |  |  | Sitting time | High | Unclear | Unclear | Unclear | Unclear | Unclear | High |
| Salmon 2008 | C-RCT | PA | Vigorous PA | Low | Low | Unclear | Unclear | High | Low | High |
| Santos 2014 | C-RCT | DPA | Total PA | Low | Low | Unclear | Low | Low | Low | Low |
| Sgambato 2019 | C-RCT | DPA | Fruit intake | Low | Low | High | Low | High | Unclear | Unclear |
|  |  |  | Water intake | Low | Low | High | Low | High | Unclear | Unclear |
|  |  |  | Total PA | Low | Low | High | Low | Low | Unclear | Unclear |
| Sichieri 2008 | C-RCT | D | Energy intake | Unclear | Low | High | Unclear | High | Low | High |
|  |  |  | Soft drink intake | Unclear | Low | High | Unclear | High | Low | High |
| Siegrist 2013 | C-RCT | DPA | MVPA | Unclear | Unclear | High | High | Unclear | Low | Unclear |
| Siegrist 2018 | C-RCT | DPA | Total PA | Unclear | Low | High | Low | Low | High | High |
|  |  |  | Soft drink intake | Unclear | Low | High | Unclear | High | High | High |
| Simon 2008 | C-RCT | PA | Leisure PA | Low | Low | High | High | High | Low | Unclear |
| Singh 2009 | C-RCT | DPA | SSB intake | Low | Low | High | Low | Low | Low | High |
| Smith 2014 | C-RCT | PA | SSB intake | Low | Low | High | Low | Low | Low | Unclear |
|  |  |  | MVPA | Low | Low | Unclear | High | Low | Low | Unclear |
| Story 2003a | RCT | DPA | Energy intake | Low | Unclear | High | Low | Unclear | Low | Unclear |
|  |  |  | Fruit and vegetable intake | Low | Unclear | High | Low | Unclear | Low | Unclear |
|  |  |  | Water intake | Low | Unclear | High | Low | Unclear | Low | Unclear |
|  |  |  | MVPA | Low | Unclear | Unclear | Low | Unclear | Low | Unclear |
| Telford 2012 | C-RCT | PA | Total PA | Unclear | Unclear | Unclear | Unclear | Unclear | Unclear | Unclear |
| TenHoor 2018 | C-RCT | PA | MVPA | Low | Unclear | Unclear | High | High | Unclear | High |
|  |  |  | Sedentary behaviour | Low | Unclear | Unclear | High | High | Unclear | High |
| Viggiano 2018 | C-RCT | DPA | Total PA | Unclear | Unclear | High | High | Unclear | High | High |
| Wang 2018 | C-RCT | DPA | MVPA | Low | Unclear | High | Low | High | Unclear | Unclear |
| Warren 2003 | RCT | D, PA, DPA | Fruit intake | Low | Unclear | High | High | Unclear | Low | High |
| Waters 2017 | C-RCT | DPA | Vegetable intake | Low | Low | High | High | High | High | High |
|  |  |  | Soft drink intake | Low | Low | High | High | High | High | High |
|  |  |  | Active games at lunch | Low | Low | High | High | High | High | High |
| Wendel 2016 | C-RCT | DPA | Total PA | Unclear | Unclear | High | Low | Unclear | High | High |
| White 2019 | RCT | DPA | Vigorous PA | Low | Unclear | Unclear | High | Unclear | Low | High |
|  |  |  | Sedentary behaviour | Low | Unclear | Unclear | High | Unclear | Low | High |
| Wilksch 2015 | C-RCT | DPA | Total PA | Unclear | Unclear | High | High | Unclear | Low | High |
| Williamson 2012 | C-RCT | DPA | Energy intake | Unclear | Unclear | Unclear | Low | Low | Low | High |
|  |  |  | Total PA | Unclear | Unclear | High | Low | Low | Low | High |
|  |  |  | Sedentary behaviour | Unclear | Unclear | High | Low | Low | Low | High |
| Xu 2015 | C-RCT | DPA | Vegetable intake | Low | Unclear | High | Low | Low | Unclear | Unclear |
|  |  |  | Soft drink intake | Low | Unclear | High | Low | Low | Unclear | Unclear |
|  |  |  | Walking | Low | Unclear | High | Low | High | Unclear | Unclear |
| Xu 2017 | C-RCT | D, PA, DPA | Energy intake | Low | Unclear | High | Low | Unclear | Low | Unclear |
|  |  |  | Vegetable intake | Low | Unclear | High | Low | Unclear | Low | Unclear |
| Zhou 2019 | C-RCT | DPA | MVPA | Unclear | Unclear | Unclear | Unclear | Low | Unclear | High |
|  |  |  | Sedentary behaviour | Unclear | Unclear | Unclear | Unclear | Low | Unclear | High |
| Zota 2016 | C-RCT | D | Fruit intake | Low | Unclear | Unclear | High | Unclear | Unclear | High |

RCT = randomised controlled trial; C-RCT = cluster-randomised controlled trial; D = diet; PA = physical activity; DPA = diet and physical activity; MVPA = moderate-vigorous physical activity; SSB = sugar-sweetened beverage
